# *WDR62* variants contribute to congenital heart disease by inhibiting cardiomyocyte proliferation

**DOI:** 10.1002/ctm2.941

**Published:** 2022-07-08

**Authors:** Lili Hao, Jing Ma, Feizhen Wu, Xiaojing Ma, Maoxiang Qian, Wei Sheng, Tizhen Yan, Ning Tang, Xin Jiang, Bowen Zhang, Deyong Xiao, Yanyan Qian, Jin Zhang, Nan Jiang, Wenhao Zhou, Weicheng Chen, Duan Ma, Guoying Huang

**Affiliations:** ^1^ Shanghai Key Laboratory of Birth Defects Children's Hospital of Fudan University Shanghai China; ^2^ Key Laboratory of Metabolism and Molecular Medicine Ministry of Education School of Basic Medical Sciences Fudan University Shanghai China; ^3^ Fudan University Shanghai Cancer Center Shanghai China; ^4^ ENT institute Department of Facial Plastic and Reconstructive Surgery Eye & ENT Hospital of Fudan University Shanghai China; ^5^ Laboratory of Epigenetics Institutes of Biomedical Sciences Fudan University Shanghai China; ^6^ Department of Medical Genetics Department of Clinical Laboratory Liuzhou Maternity and Child Healthcare Hospital Liuzhou Guangxi China; ^7^ Medical Laboratory of Nantong ZhongKe Nantong Jiangsu; ^8^ Research Unit of Early Intervention of Genetically Related Childhood Cardiovascular Diseases Chinese Academy of Medical Sciences Shanghai China

**Keywords:** cardiomyocyte proliferation, cell cycle, congenital heart disease, spindle assembly, the WDR62 gene

## Abstract

**Background:**

Congenital heart disease (CHD) is the most common birth defect and has high heritability. Although some susceptibility genes have been identified, the genetic basis underlying the majority of CHD cases is still undefined.

**Methods:**

A total of 1320 unrelated CHD patients were enrolled in our study. Exome‐wide association analysis between 37 tetralogy of Fallot (TOF) patients and 208 Han Chinese controls from the 1000 Genomes Project was performed to identify the novel candidate gene WD repeat‐containing protein 62 (*WDR62*). *WDR62* variants were searched in another expanded set of 200 TOF patients by Sanger sequencing. Rescue experiments in zebrafish were conducted to observe the effects of *WDR62* variants. The roles of WDR62 in heart development were examined in mouse models with *Wdr62* deficiency. *WDR62* variants were investigated in an additional 1083 CHD patients with similar heart phenotypes to knockout mice by multiplex PCR‐targeting sequencing. The cellular phenotypes of *WDR62* deficiency and variants were tested in cardiomyocytes, and the molecular mechanisms were preliminarily explored by RNA‐seq and co‐immunoprecipitation.

**Results:**

Seven *WDR62* coding variants were identified in the 237 TOF patients and all were indicated to be loss of function variants. A total of 25 coding and 22 non‐coding *WDR62* variants were identified in 80 (6%) of the 1320 CHD cases sequenced, with a higher proportion of *WDR62* variation (8%) found in the ventricular septal defect (VSD) cohort. *WDR62* deficiency resulted in a series of heart defects affecting the outflow tract and right ventricle in mouse models, including VSD as the major abnormality. Cell cycle arrest and an increased number of cells with multipolar spindles that inhibited proliferation were observed in cardiomyocytes with variants or knockdown of *WDR62*. *WDR62* deficiency weakened the association between WDR62 and the cell cycle‐regulated kinase AURKA on spindle poles, reduced the phosphorylation of AURKA, and decreased expression of target genes related to cell cycle and spindle assembly shared by WDR62 and AURKA.

**Conclusions:**

*WDR62* was identified as a novel susceptibility gene for CHD with high variant frequency. WDR62 was shown to participate in the cardiac development by affecting spindle assembly and cell cycle pathway in cardiomyocytes.

## Graphical abstract

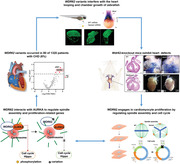

## 1 INTRODUCTION

Congenital heart disease (CHD) is the most common birth defect worldwide, affecting ∼1% of newborn children, with the highest prevalence being in Asia.^1^, ^2^ Cardiac development involves many complicated biological processes, including proliferation, differentiation, and apoptosis of multiple cell lineages of myocardial, endothelial, mesenchymal, neural crest, and epicardial origins.^3^, ^4^ Abnormalities in any of these processes can lead to various types of CHD. The five most common subtypes (except for bicuspid aortic valve) are atrial septal defect (ASD), ventricular septal defect (VSD), patent ductus arteriosus (PDA), coarctation of the aorta (CoA), and tetralogy of Fallot (TOF).^1^, ^5^, ^6^ CHD is also classified as syndromic or nonsyndromic depending on whether or not abnormalities are present in other organs, with nonsyndromic CHD accounting for 80% of all cases.^7^, ^8^ Although surgical management decreases the death rate of those with CHD, patients are still at high risk for serious complications such as heart failure and sudden death.^9^

Genetic defects play an important role in the occurrence and development of CHD.^6^, ^10^ The mutation of single gene, such as *NKX2.5*, *JAG1*, and *TBX20*, were firstly identified by linkage analysis in pedigrees of CHD with Mendelian inheritance patterns.^8^, ^11^ These genes can only explain the etiology of about 4% CHD cases.^12^ In fact, most CHD is sporadic and the genetic causes are often involved in co‐action of multiple genes.^6^, ^8^ With the help of next‐generation sequencing technology, some susceptibility genes for CHD, such as *SOX17*,^13^ *GLI1*,^14^ and *MYH6* ^15^ were detected in large sporadic cohorts. However, the genetic etiology of 70% of CHD cases remains unknown due to high heterogeneity and incomplete penetrance.^11^, ^16^

Here, we found high frequency variants in *WDR62* (WD repeat‐containing protein 62) by sequencing analysis in a large CHD cohort. WDR62 is a microtubule and centrosome‐associated protein required in centriole duplication and astral microtubule assembly.^17^, ^18^, ^19^ While it has not been reported to function in heart development, we found that malformation of the heart and dysregulation of cardiomyocyte proliferation were associated with WDR62 deficiency both in vivo and in vitro. These data suggest that WDR62 is a susceptibility gene for CHD with high frequency variants.

## 2 METHODS

### 2.1 Clinical subjects

We only focused on the key genes involved in cardiomyogenesis, so the 1320 patients (age 0–216 months) with nonsyndromic CHD were enrolled. CHD subtypes included isolate VSD, pulmonary atresia combined with VSD (PA+VSD), double outlet right ventricle combined with PS (DORV+PS) and right ventricular dysplasia (RVD) (Table 1). The patients were from Children's Hospital of Fudan University in Shanghai, China, and were diagnosed by echocardiography using published diagnostic criteria.^20^ They had no extra physical abnormalities.

**TABLE 1:** Statistics of sex and age of 1320 patients with CHD

**Subtype**	**Cases (number)**	**Median age at diagnosis**	**Gender No**.	
		**(IQR), months**	**Male**	**Female**
TOF cases for case‐control analysis				
TOF	With variants (4)	12 (7.5–23.25)	3	1
	All (37)	10 (7.75–36.25)	18	19
TOF cases for Sanger sequencing				
TOF	With variants (2)	12, 35	1	1
	All (200)	12 (6–32)	125	75
Cases for multiplex PCR‐targeting sequencing				
TOF	With variants (8)	9.5 (6–24)	4	4
	All (234)	8 (6–18)	142	96
VSD	With variants (55)	7 (4–20.75)	31	24
	All (718)	12 (5–36)	405	313
PA+VSD	With variants (3)	10, 10, 32	2	1
	All (55)	12 (7–34)	30	25
DORV+PS	With variants (2)	16, 36	1	1
	All (19)	36 (7.5–54)	10	9
RVD	With variants (6)	10 (5.75–16.5)	5	1
	All (57)	17(5–42)	27	25
Total	With variants (80)	9 (5–20)	47	33
	All (1320)	10 (5–36)	760	560

Abbreviation: IQR, interquartile range.

Thirty‐seven TOF patients were initially collected for whole‐exome sequencing (WES). All the 32 exons and splice sites of *WDR62* were validated by Sanger sequencing in another 200 TOF individuals. Further multiplex PCR‐targeting sequencing for all exons, splice sites, and regulatory regions of *WDR62* was applied in additional 1083 CHD patients.

### 2.2 Ethics statement

The study conformed to the tenets of the Declaration of Helsinki (1983 Revision). Studies of human subjects were permitted by the Medical Ethics Committee of Children Hospital of Fudan University, the approval number is: 2016–121. All mice and zebrafish procedures were approved and monitored by the Research Ethics Committee of the School of Basic Medical Sciences, Fudan University, China. The approval numbers are 20160520–3 and 20150119‐002.

### 2.3 WES and bioinformatics analysis

Genomic DNA (gDNA) from the peripheral blood of 37 childhood patients with TOF was extracted using a QIAamp DNA Blood Mini Kit (Qiagen). The SureSelect Human All Exon V6 kit (Agilent Technologies) was used for capture and library preparation. Paired‐end sequencing with a 150 bp read length was performed using the Illumina NovaSeq platform with a coverage of 20 times for 82–99% of the targeted exomic regions. Sequence reads were aligned to the human genome reference sequence (GRCh37) using Burrows–Wheeler Aligner (BWA) MEM algorithm (version 0.7.17). Indel realignment, base and quality score recalibrations, and removal of PCR duplicates from the resultant Binary Alignment Map (BAM) files were performed according to the Genome Analysis Toolkit (GATK)^21^, ^22^ (version 3.7) and Picard tools (version 2.18.25). For variant calling, the germline variants were filtered with variant quality score recalibration (VQSR) by setting truth sensitivity filter level at 99. Sequence variants with one of the following features were further excluded: (a) the read depth < 20; (b) the variant allele frequency < 30%; (c) the genotype quality (GQ ) < 20.

Sequence variants were functionally annotated by the ANNOVAR program,^23^ with annotation databases, including the Reference Sequence (RefSeq) collection, the Combined Annotation Dependent Depletion (CADD, version 1.3),^24^ the Sorting Intolerant From Tolerant (SIFT),^25^ the Polymorphism Phenotyping 2 (Polyphen2),^26^ the Genome Aggregation Database (GnomAD) exome, and genome collections (version 2.0.1). Non‐coding, synonymous coding variants, or common variants with ≥ 0.1% population frequency in the gnomAD exome collections were excluded from further consideration for this study. The rare non‐silent variants with strong in‐silico evidence supporting possible pathogenicity (i.e., CADD Phred score > 20 [top 1% deleterious] without tolerated/benign status by both SIFT and Polyphen2) were included as the final probably risk variants for further comparison.

### 2.4 Analysis of sequence variants from 1000 Genomes Project (1000 G) and gene‐based rare variant association tests

A total of 208 unrelated Han Chinese (103 Han Chinese in Beijing, China [CHB] and 105 Southern Han Chinese [CHS]) genotyped by whole exome/genome sequencing by phase 3 of 1000 G^27^ were included in this analysis. The files in variant call format (VCF) from version 5 data release including genotype data for 2504 unrelated individuals were downloaded by ftp (ftp://ftp‐trace.ncbi.nih.gov/1000genomes/ftp/release/20130502). The genotype data in the common regions covered with the Agilent SureSelect kit for these 208 unrelated Han Chinese were extracted using the SelectVariants function of GATK. To determine the significance of our findings in pediatric patients with TOF compared to control, we applied the same annotation and filtering process to classify the probably risk variants in this control dataset (refer to the WES method part). Principal components analysis (PCA) was performed to demonstrate that the results were not affected by ethnicity.

We performed gene‐based rare variant association tests, and a total of 4013 genes with at least one rare and probably risk variant in TOF cases were analysed by comparing the variant frequencies between TOF cases and controls with Fisher's exact test. For all tested genes with more than two rare and probably risk variants in TOF cases, we estimated a false discovery rate < 10% with a nominal *p*‐value < 0.005, using Benjamini–Hochberg procedure.

### 2.5 *zwdr62* knocked‐down zebrafish and rescue study

Morpholinos (MOs) were obtained from Gene Tools and were dissolved in nuclease‐free water (Thermo Fisher). Then 8 ng of MOs were injected into single cell stage embryos of Tg (cmcl2: GFP). The sequence of the splice‐inhibiting MO complementary to Exon/intron 26 (MO‐Ex26) of *zwdr62* was 5′‐AAAAGCTGATGTTGACTCACCGGAA‐3′. The standard control (Scon‐MO) was 5′‐CCTCTTACCTCAGTTACAATTTATA‐3′.

A plasmid containing full‐length human *WDR62* cDNA (NM_173636) was purchased from OriGene (OriGene Technologies). *WDR62* cDNA was then cloned into the EcoRI and XbaI sites of the pCS2+ vector. The 7 variants (c.G189T, c.A875C, c.C1441T, c.C1669T, c.G1796C, c.G2418A, and c.G3287A) were created, respectively, using the KOD‐Plus Mutagenesis Kit (Toyobo) according to the manufacturer's instructions. All the expression plasmids were fully sequenced.

Capped and poly (A) tailed mRNA of human *WDR62* (*hWDR62*) and variants were synthesized in vitro using the mMessage mMachine SP6 Kit (Ambion). All injections were performed as at least three separate experiments. For rescue experiments, mRNA (150 ng) and MOs (8 ng) were mixed and co‐injected into embryos of Tg (cmcl2: GFP) at the single cell stage.

The efficiencies of MO‐Ex26 and mRNA overexpression were verified in injected embryos collected at 48 hpf by quantitative real‐time PCR (qPCR). The primers were as follows: *zwdr62*‐MO‐F, 5′‐CAGAGAGCAACTTCCTCAATCC‐3′; *zwdr62*‐MO‐R, 5′‐TGATGTTGACTCACCGGAAAC‐3′; *hWdr62*‐Q‐F, 5′‐AGGGACAGACAGCCAGTATT‐3′; *hWdr62*‐Q‐R, 5′‐GACACCCTGAGGTAGGAGTC‐3′. Fifty embryos with cardiac GFP at 48 hpf from each injection group were observed for cardiac morphology using a Leica M205C microscope.

### 2.6 Proliferation assays

Zebrafish embryos were collected at 48 hpf to perform whole‐mount immunofluorescence. After fixing in 4% paraformaldehyde (PFA) at 4°C overnight, digestion using Collagenase, Type II (Life technologies), and blocking at room temperature, the embryos were incubated with the rabbit anti‐Histone H3 (phospho S10) (ab5176, Abcam) and mouse anti‐BrdU (sc‐32323, Santa Cruz Biotechnology). For BrdU assay, embryos were exposed to BrdU for 6 h before collection.

For tissue sections of mouse hearts, the proliferation of cardiomyocytes was analysed by immunohistochemical staining using rabbit anti‐Ki67 (ab15580, Abcam) and rabbit anti‐Histone H3 (phospho S10) (ab5176, Abcam). To identify the positivity of the immune‐stained sections, negative controls (NCs) were set that the slices from the same batch were only incubated with isotype matched secondary antibodies without binding with specific primary antibodies.

### 2.7 Analysis of potential genes co‐regulated by WDR62 and AURKA

Downregulated genes resulted from AURKA deficiency were from Gene Expression Omnibus (GEO), accession numbers: GSE57810 and GSE23541. Overlap of genes among ‘DOWN group’, ‘CORE genes’ and downregulated genes caused by AURKA knockdown were displayed through venn diagram drawn by Jvenn.^28^ HL‐1 cells were treated with 500 nmol/L of Aurora A kinase inhibitor MLN8237 (Selleck) for 24 h. Afterward, the cells were harvested and fixed with 70% ice‐cold ethanol at −20°C for at least overnight.

### 2.8 Statistical analysis

**FIGURE 1:**
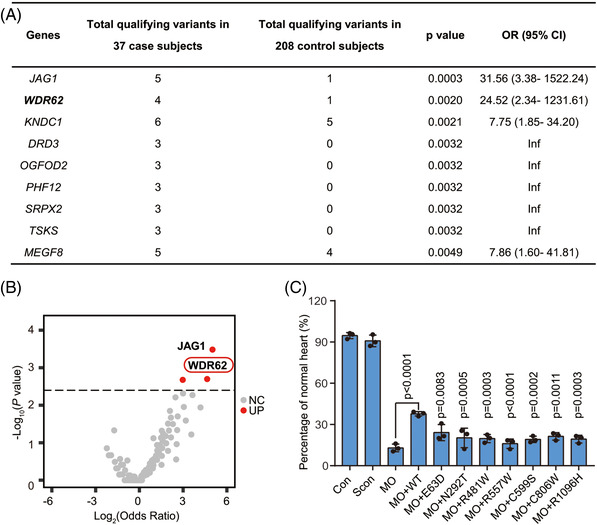
Significant association of WDR62 with TOF and the harmful effects of WDR62 variants. (A) All candidate genes with nominal *p*‐value < 0.005 and a false discovery rate < 10% using the Benjamini–Hochberg procedure. CI = confidence interval. (B) Volcano plot showing the results of an association test of probably pathogenic variants in 4013 genes using 37 TOF cases and 208 controls. The non‐significant genes are represented in grey, and the significant genes are represented in red (*p*‐value < 0.005). (C) The variants of *hWDR62* failed to rescue the cardiac malformation of *wdr62*‐MO zebrafish. Approximately 95% of WT control (Con) and 91% of standard control (injected with control MO, Scon) exhibited normal cardiac formation, which was significantly higher than *wdr62*‐MO (13%). The average normal heart ratio was ∼20% when *hWDR62* variant mRNA was co‐injected with MO, whereas the ratio of normal hearts was increased to 38% with the co‐injection of wild type *hWDR62* mRNA with MO. In each trial, at least 36 embryos from each group were analysed (Con, *n* = 261; Scon, *n* = 65; MO, *n* = 70; MO+WT, *n* = 70; MO+E63D, *n* = 45; MO+N292T, *n *= 50; MO+R481W, *n *= 47; MO+R557W, *n* = 57; MO+C599S, *n* = 67; MO+C806W, *n* = 79; MO+R1096H, *n* = 47; n is the average number of embryos in three experiments). The percentage of zebrafish with normal heart formation is presented as the mean ± standard deviation (SD) of three experiments. Statistical significance was assessed using one‐way ANOVA and post hoc Tukey–Kramer test. The *p*‐values indicate comparison versus MO+WT unless marked. Blue colour indicates the proportion of embryos with normal hearts

**FIGURE 2:**
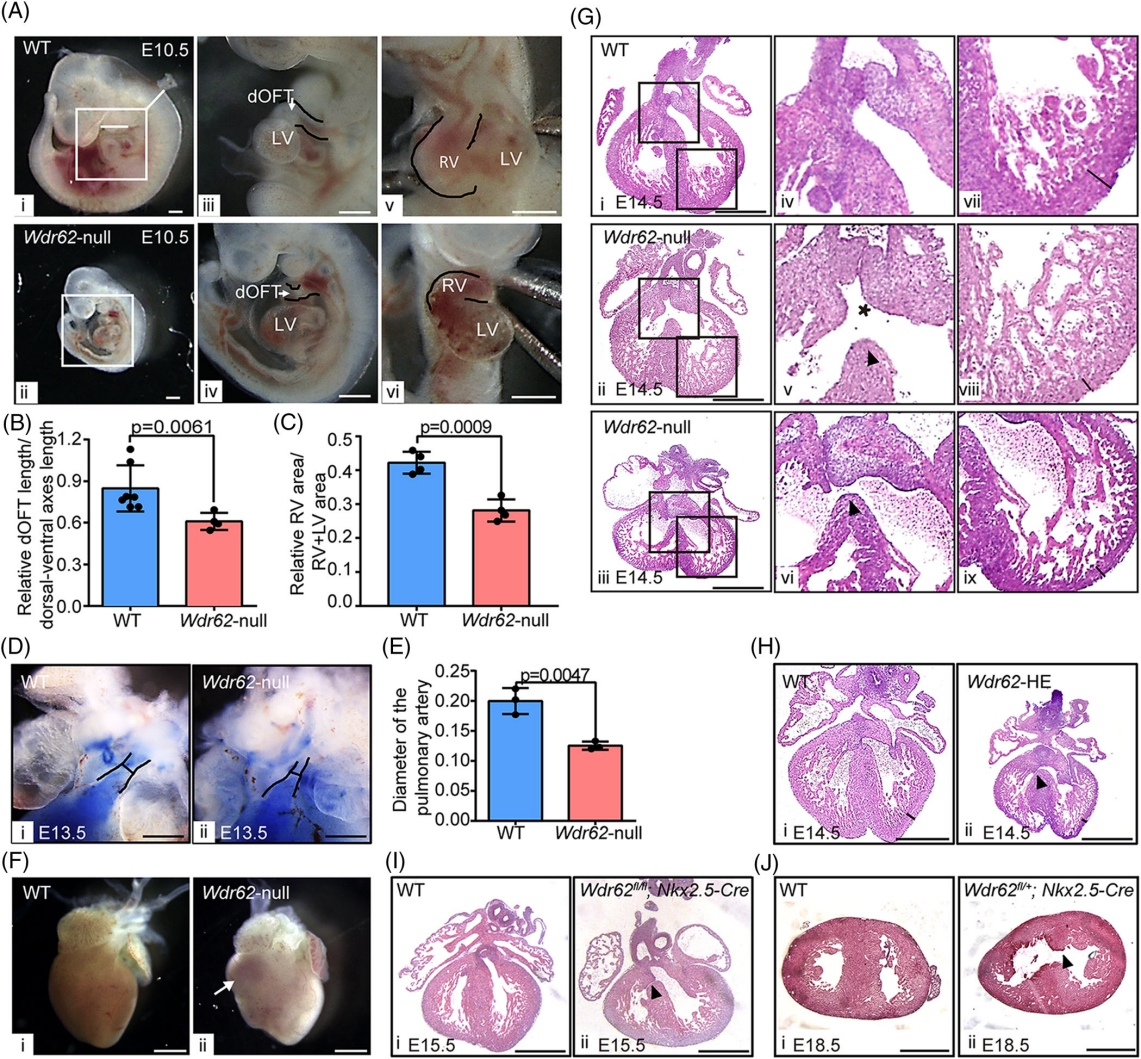
Heart abnormalities of mice with Wdr62 deficiency. (A) Compared with WT heart at E10.5, *Wdr62*‐null hearts exhibited smaller RV and shorter distal OFT (dOFT). Details of abnormalities in (i, ii) are magnified in (iii–vi). The dOFT length and dorsal‐ventral axes lengths are indicated by white lines in (i) and the dOFT is indicated by black outline in (iii and iv). The size of RV is indicated by black outline in (v and vi). Scale bar = 0.5 mm. (B) Quantification analysis of dOFT length. The data are expressed as mean ± SD. (WT, n = 7; *Wdr62*‐null, n = 4). (C) Quantification analysis of RV area. The data are expressed as mean ± SD. (WT, n = 4; *Wdr62*‐null, n = 4). (D) Pulmonary stenosis in *Wdr62*‐null embryos (ii). Trypan blue was injected into the heart to show the pulmonary artery. The outline and diameter of the pulmonary artery are indicated by black lines. Scale bar = 0.5 mm. (E). Quantification analysis of the length of pulmonary artery diameter. Data are presented as mean ± SD. (WT, n = 3; *Wdr62*‐null, *n* = 3). (F) Right ventricular hypertrophy in *Wdr62*‐null mice is indicated by the white arrow (ii). Scale bar = 1 mm. (G) Hematoxylin‐eosin (H&E) staining of heart sections in WT and *Wdr62*‐null mice at E14.5. Details of abnormalities in (i–iii) are magnified in (iv–ix). Scale bar = 0.5 mm. (H) H&E staining results of heart sections in WT and Wdr62‐HE at E14.5. Scale bar = 0.2 mm. (I) H&E staining results of heart sections in WT and *Wdr62^fl/fl^; Nkx2.5‐Cre* at E15.5. Scale bar = 0.2 mm. (J) Cross‐section of WT and *Wdr62^fl/+^; Nkx2.5‐Cre* at E18.5. Scale bar = 0.5 mm. For (G–J), VSD is indicated by black arrows, OA is indicated by the star, and thinning of ventricular wall is indicated by black lines

All values were expressed as mean ± standard deviations (SD). Statistical tests were performed using GraphPad Prism Software (version 7). In Figures 2B,C,E and 4E,F and Supplementary Figures S5B, S6A,B and S8E, the differences between two groups were analysed by two‐tailed unpaired Student's *t* test with/without Welch's correction or Mann–Whitney *U* test. In Figures 1, 4 and 6C and SupplementaryFigures S3A, S5D, S7A and S8B, the differences among multiple groups were analysed using Kruskal–Wallis one‐way ANOVA and post hoc Tukey–Kramer test. In Figure 1A, Supplementary Tables S1,3,4,6,15, *p*‐values were calculated using two‐sided Fisher's exact test with/without Benjamini–Hochberg correction. In Figure S7B, the linear correlation was tested and Pearson or Spearman correlations were applied. All experiments were repeated more than twice. A *p*‐value < 0.050 was regarded as statistically significant.

### 2.9 Data availability

The additional methods used in our study are provided in the supplemental information.

## 3 RESULTS

### 3.1 *WDR62* is a candidate susceptibility gene of CHD

TOF is the most complex and serious among the major subtypes of CHD with abnormal outflow tract (OFT) and its exact genetic basis is far from clear.^6^, ^29^, ^30^, ^31^ Here, WES and case‐control analysis were performed in 37 nonsyndromic TOF individuals and 208 matched Han Chinese controls from 1000 G (Supplementary Figure S1). To determine the significantly enriched genes in TOF patients compared to control, we applied the same annotation and filtering process to classify the ‘candidate risk’ variants (minor allele frequency < 0.1%, CADD Phred score > 20 without tolerated/benign status by both SIFT and Polyphen2) in these two datasets. A total of nine genes were selected with a Fisher's exact test *p*‐value < 0.005 under a false discovery rate < 10% using Benjamini–Hochberg procedure, including one reported TOF‐associated gene, *JAG1* ^32^ on the top of list (Figures 1A,B and Supplementary Table S1). We also found that *WDR62* may be associated with TOF; *WDR62* had the second lowest *p*‐value (0.002) and a relatively higher odds ratio (OR; 95% confidence interval [CI] = 24.52 [2.34‐1231.61]) (Figure 1A, Supplementary Table S1), and its role in heart development was unknown. Four heterozygous variants (p.E63D, p.N292T, p.R481W, and p.R557W) predicted to be damaging were identified in four of the 37 TOF cases (Supplementary Table S2). We then identified three additional variants with predicted damaging effects in an expanded sample of another 200 TOF patients by *WDR62* Sanger sequencing (Supplementary Table S2). Further tests were conducted to confirm the function of this gene and the harmfulness of its variants.

We firstly chose zebrafish, an efficient and rapid model of heart development, to study the effects of the seven variants observed in TOF patients. Abundant expression of *wdr62* in the zebrafish heart was confirmed by whole mount in situ hybridization (Supplementary Figure S2A). *wdr62* knockdown by morpholinos (*wdr62*‐MO) caused 80% of zebrafish showing heart defects including smaller chamber size and abnormal looping such as sinistral loop and no loop by morphological observation (Supplementary Figure S2B, Supplementary Table S3). Narrowed heart cavity and thinner chamber wall were also observed through histologic sections (Supplementary Figure S2C). The dimensional reconstructed heart of *wdr62*‐MO showed insufficient torsion angle of OFT (Supplementary Figure S2D). Rescue experiment was performed by co‐injecting *wdr62*‐MO and human mature *WDR62* (hWDR62) mRNA (wild type [WT] or one of the seven variant sites). We found that the WT hWDR62 mRNA could rescue ∼28% of abnormal cardiac phenotypes, but variants could only rescue 7–9% (Figure 1C). These results suggest that the seven variants we identified were loss‐of‐function alleles and had harmful effects on cardiac development.

### 3.2 *Wdr62*‐deficient mice exhibited cardiac defects

Considering the large anatomic and physiologic discrepancies between the heart of zebrafish and that of mammals, we further clarified the effects of WDR62 on heart development in mice. The spatiotemporal expression pattern analysis of mouse heart indicated that *Wdr62* mRNA exhibited the highest level at E9.5 and tapered off until birth, increased again on P0 and then decreased (Supplementary Figure S3A). Immunostaining results showed that WDR62 was abundantly expressed in the myocardium of OFT and the right ventricle (RV) (Supplementary Figures S3B–C).

Then, a mouse model with germline inactivated *Wdr62* was constructed by crossing *Wdr62*‐floxed mice to *CMV‐Cre* mice (Supplementary Figures S4A–D), and the absence of the *Wdr62* in whole embryos of homozygous *Wdr62* knockout (*Wdr62*‐null) mice was confirmed (Supplementary Figure S4E). Analysis of gross external morphology revealed that over 80% *Wdr62*‐null mice exhibited a range of abnormalities including developmental retardation, coloboma or microphthalmia, microcephaly, and infertility (Supplementary Figures S4F, Supplementary Table S4). Moreover, a sub‐Mendelian ratio was observed in *Wdr62*‐null mice from E13, suggesting embryonic death (Supplementary Table S5). The early deformities of shorter OFT and smaller RV began to appear at E9.5‐E10.5 in *Wdr62*‐null embryos (Figures 2A–C, S5A‐B). Major changes in the appearance of the heart from E12.5 to P0 were as follows: Cardiac growth delay (45%), great vessel alignment defects (50%), shorted OFT (42%) and narrowed RV outflow tract (RVOT) including pulmonary stenosis (PS; 58%) (Figures 2D–E, S5C, Supplementary Table S6). Right ventricular hypertrophy could also be observed in some P1 *Wdr62*‐null mice (Figure 2F). At E14.5 of completed heart separation, the internal structure defects were analysed (Figure 2G). Notably, VSD with different severity was detected in all *Wdr62*‐null mice performing histological examination. Overriding aorta and thinner compact layer of ventricular myocardium were also observed (Figure 2G). In general, 75% of *Wdr62*‐null mice showed heart defects (Supplementary Table S6). In addition, the incidence of cardiac abnormalities increased significantly in heterozygous global knockout (HE) mice when compared with WT (Supplementary Table S6). Moreover, mild VSD was detected in 37.5% of HE mice and 20% of cardiomyocyte‐specific knockout mice (crossing *Wdr62*‐floxed mice to *Nkx2.5‐Cre* mice to generate *Wdr62^fl/fl or fl/+^; Nkx2.5‐Cre*) (Figures 2H–J) Taken together, knocking out *Wdr62* caused not only TOF phenotypes, but also other defects associated with malformation of RV and OFT, especially VSD.

### 3.3 High variant frequency of *WDR62* was found in CHD patients

**FIGURE 3:**
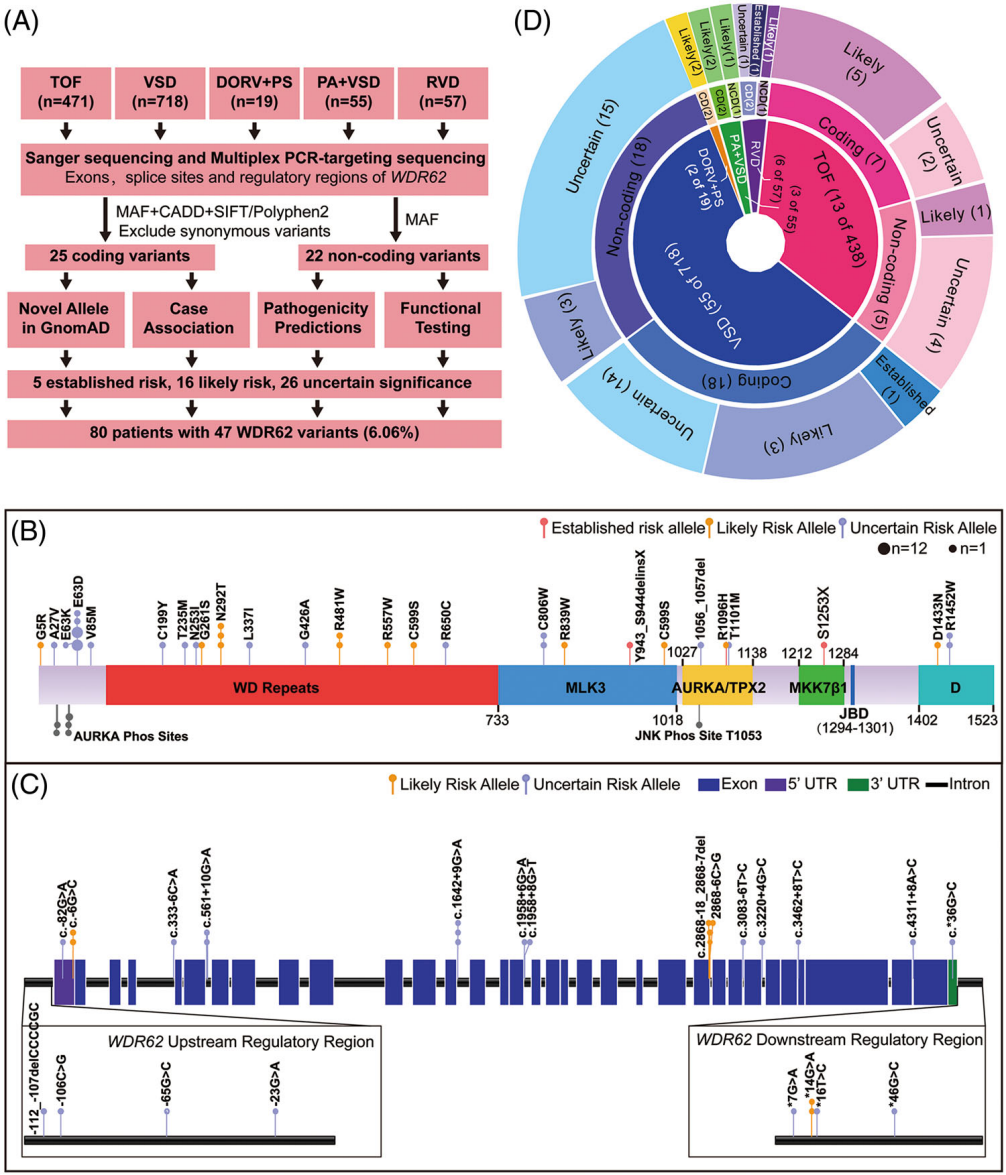
Analysis and distribution of *WDR62* variants. (A) Illustration for the identification of *WDR62* variants as risk alleles for CHD. (B) Protein domain plot of WDR62^48^ and 24 coding variants. (C) Genomic structure of *WDR62*, including 120 bp upstream of the transcription start site, 80 bp downstream of the transcriptional termination site, exons, and introns. Intronic regions are at 1:20 scale of the exons. The two boxes below show the amplification of the upstream and downstream sections. All identified *WDR62* noncoding variants are indicated in the corresponding position. In (B and C), all variants are coloured according to the classification of risk alleles. Each small circle represents a unique individual who carries the indicated variant. The large circle represents 10 cases carrying the variant. (D) Statistical overview of *WDR62* variants. The inner layer presents the distribution of *WDR62* variants in five CHD subtypes, the number of variant carriers and the total number of patients in four groups are in brackets; the middle is the number of coding (CD) and noncoding (NCD) variants in different patient groups; the outer shows the risk classification of *WDR62* variants. The digit in brackets in middle and outer layers indicates variant number

WDR62 deficiency was observed to be involved in multiple abnormalities of cardiac development in mice and zebrafish, and to determine the degree of association between WDR62 variants and CHD, we recruited additional human CHD cases corresponding to the types and frequency of heart defects observed in knockout mice: Isolated VSD, TOF, RVD, and another two types of OFT defects—PA+VSD and DORV+PS. The *WDR62* variants in these 1083 CHD cases were analysed through multiplex PCR‐targeting sequencing (Figure 3A, Table 1). Combined with the sequencing results of the 237 previous TOF cases, for a total of 1320 cases, 25 candidate risk variants were identified in 55 patients (Figure 3B, Supplementary Tables S7, S8). These included 22 missense, two nonsense, and one non‐frameshift deletion in the *WDR62* coding region (Supplementary Table S7). Six variants were absent in all searched standard population databases (1000 G, Exome Variant Server [ESP6500], the Exome Aggregation Consortium [ExAC] and GnomAD) (Supplementary Table S9). The missense variants were mainly located in the conserved *N*‐terminal half of WDR62, which contains WD repeats (16 of 733 amino acid [aa] vs. 6 of 790 aa; OR [CI] = 2.92 [1.18–7.15], *p* = 0.0029) (Figure 3B, Supplementary Tables S10). In addition, 22 rare variants located in the non‐coding region were identified in 26 patients, including 11 variants located in introns, 3 in untranslated regions (UTRs), and 8 in upstream or downstream regulatory regions (Figure 3C, Supplementary Tables S8, S11). Fifteen were predicted to be damaging by impairing splicing consensus, microRNA binding, or regulatory DNA elements (Supplementary Tables S12‐S14). To further analyse the enrichment of individual variants in CHD, we also performed case‐control association studies and found that the prevalence of nine variants was significantly increased in CHD compared with gnomAD allele counts for East Asians (Supplementary Table S15). Approximately 6% (80/1320) of CHD patients in our study had *WDR62* variants. A higher ratio of patients with *WDR62* variants (8% of 718 patients) was found in those with VSD (Figure 3D).

We classified 47 variants according to the disease causality evaluated by the guidelines of the American College of Medical Genetics and Genomics and the Association for Molecular Pathology (ACMG–AMP)^33^ as described in Methods (Supplementary Table S16). Considering all variants were from sporadic groups, we used the terms ‘established risk’ and ‘likely risk’ rather than ‘pathogenic’ and ‘likely pathogenic’ to classify variants as the ACMG suggests.^33^ Fifteen variants were considered to be high risk including two ‘established risk’ and 13 ‘likely risk’ alleles, which together accounted for 32% of all variants (Figure 3D, Supplementary Tables S17, S18). Other variants were rated as ‘uncertain risk’ alleles. Given that the seven variants (p.E63D, p.N292T, p.R481W, p.R557W, p.C599S, p.C806W, and p.R1096H) had harmful effects validated in zebrafish, and many of them were identified in both VSD and TOF cases, these variants were selected for further functional analysis.

### 3.4 WDR62‐deficiency led to spindle defects and reduced proliferation in cardiomyocytes

**FIGURE 4:**
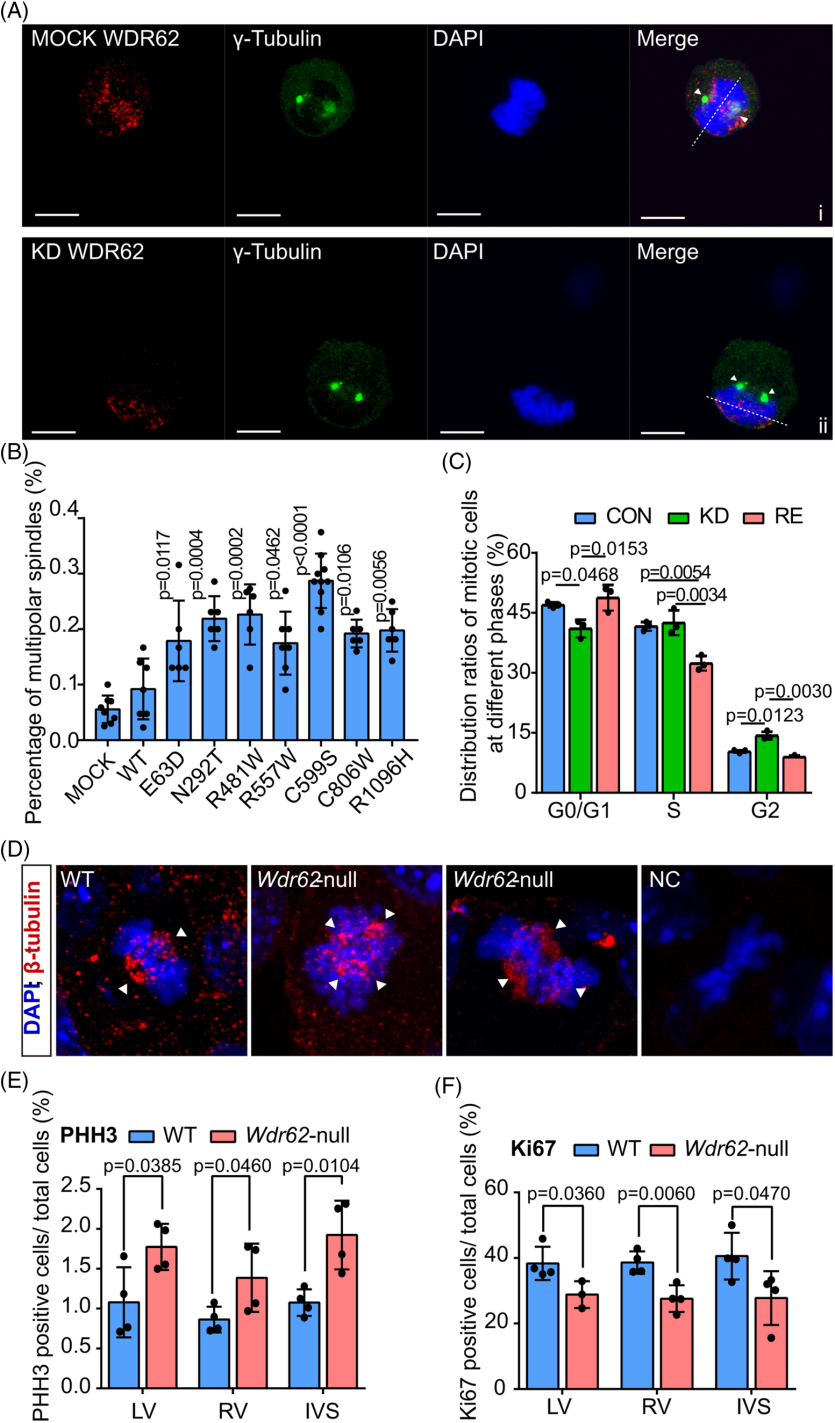
Spindle defects and cell cycle arrest observed in cardiomyocytes with WDR62‐deficiency. (A) WDR62‐WT co‐localized with spindle poles in HL‐1. The location of spindle poles changed from both sides of the chromosome plane to the same side of the chromosome plane in the WDR62 knockdown (the second line). Spindle poles are indicated with arrows. Chromosome planes are indicated by the dotted line. HL‐1 cells were stained to visualize chromosomes (DAPI; in blue), spindle poles (γ‐tubulin; in green) and WDR62 (WDR62; in red). Scale bar = 10 μm. (B) When *WDR62* variants were overexpressed, the percentage of HL‐1 cells with abnormal spindles was higher than in HL‐1 cells overexpressing empty vector (MOCK) or *WDR62*‐WT. The percentage of abnormal spindles was calculated as the number of cells with abnormal spindles out of all mitotic cells. The data are expressed as mean ± SD. (MOCK, *n* = 8; WT, *n* = 7; E63D, *n* = 6; N292T, *n* = 6; R481W, *n* = 6; R557W, *n* = 7; C599S, *n* = 10; C806W, *n* = 6; R1096H, *n* = 6). *p*‐value indicates comparison with WT. (C) Cell cycle distribution analysis of CON, KD, and RE cells. The data are expressed as mean ± SD of three experiments. (D) Multipolar spindles in cardiomyocytes observed in histologic sections of *Wdr62*‐null mice at E14.5. NC group were slices treated in the same way without incubating with specific primary antibodies. Arrows indicate spindles. (E, F) Quantification of Ki67 (E) and PHH3 (F) positive cardiomyocytes in the left ventricle (LV), right ventricle (RV), and interventricular septum (IVS) of mice at E14.5. The data are expressed as mean ± SD. (WT, *n* = 4; *Wdr62*‐null, *n* = 4)

WDR62 is involved in spindle formation and mitotic progression, which may affect the heart development by making impacts on cell cycle and proliferation. Therefore, in vitro functions of WDR62 were investigated in HL‐1, an murine cardiomyocyte cell line with proliferation ability.^34^ WDR62 was primarily detected in spindle poles and microtubule during mitosis (Figure 4A). When WDR62 was knocked down by CRISPR‐Cas9 (KD) (Supplementary Figures S6A‐C), the orientation of spindle poles was abnormal, with distribution along chromosome one‐sided (Figure 4A). We also observed a higher proportion of cells with monopolar or multipolar (>2) spindles in KD cells, which can be rescued by overexpressing WDR62‐WT in KD (RE cell line) (Supplementary Figures S6D, S6E). The monopolar or multipolar spindles were defined as ‘abnormal spindles’. Compared with cells overexpressing WDR62‐WT, the number of cells with abnormal spindles was significantly increased as WDR62 variants were overexpressed (Figures 4B, Supplementary Figure S6F). Meanwhile, cell cycle distribution analysis showed WDR62‐KD exhibited a significant increase in G2/M‐phase content, accompanied by a corresponding reduction in the G0/G1‐phase, suggesting cell cycle arrest, which can also be rescued in RE cell line (Figure 4C).

Correspondingly, cardiomyocytes with multipolar spindles were observed in the interventricular septum of *Wdr62*‐null but not in that of WT embryos (Figure 4D). To determine whether the spindle defects affect proliferation of cardiomyocytes endogenously, we performed immunostaining of pan‐proliferative marker Ki67 as well as mitotic phase marker phospho‐histone H3 (PHH3) in *Wdr62*‐null mice. Significantly increased PHH3 labelling and reduced Ki67 labelling were found in the left and right ventricles and the interventricular septum of *Wdr62*‐null embryonic hearts (Figure 4E–F, Supplementary Figure S6G), indicating decreased proliferation and mitosis arrest. However, no significant difference in apoptotic cells was detected (data not shown).

### 3.5 WDR62 engaged in cardiomyocyte proliferation by interacting with AURKA and affecting proliferative pathways

**FIGURE 5:**
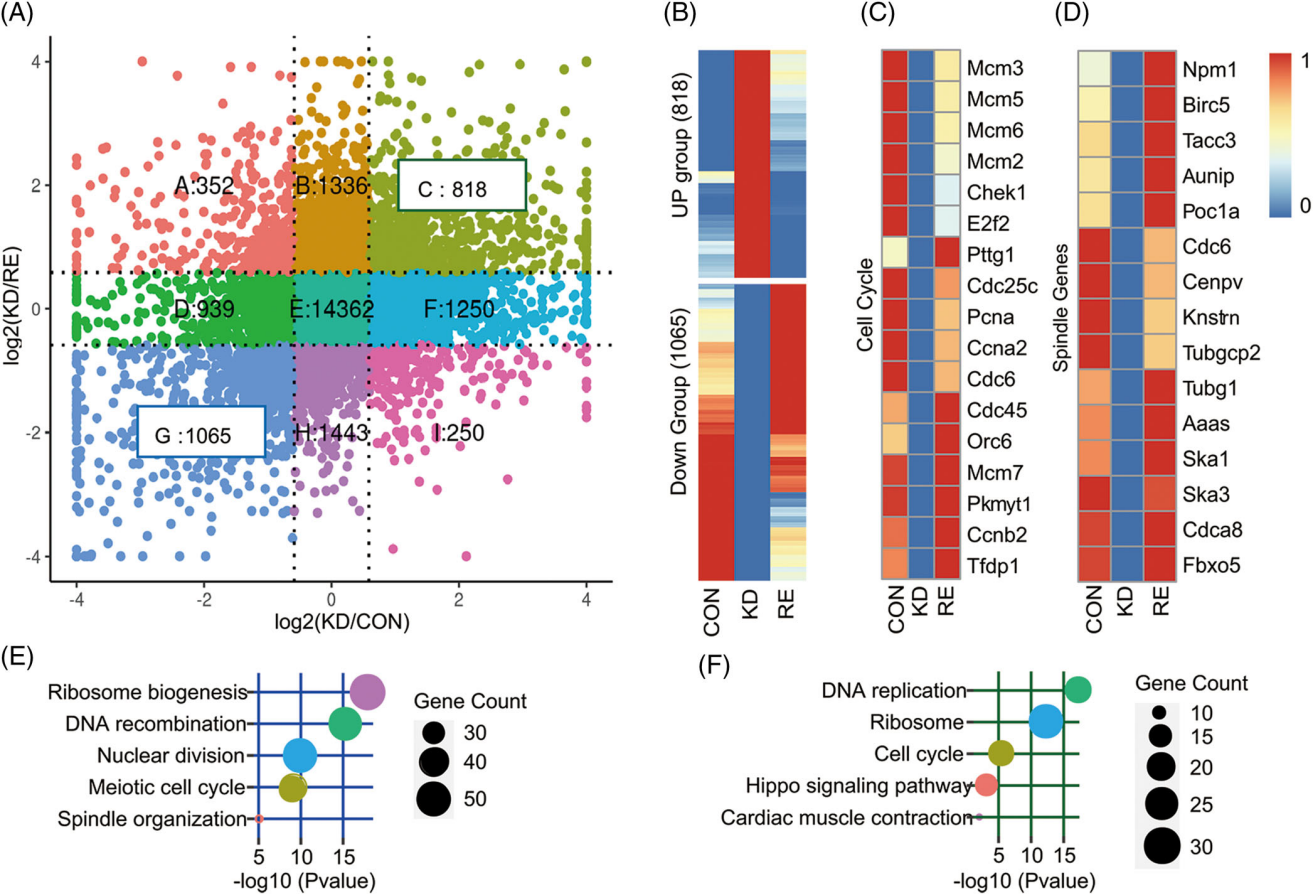
WDR62‐associated cardiomyocyte proliferative pathways. (A) Differential gene expression in KD/CON and KD/RE. RE was established by overexpressing *hWDR62* in KD. The x‐axis and y‐axis are log2 fold‐change of each gene in KD/CON and KD/RE, respectively. The dotted lines, representing log2 fold‐change = 1.5, categorize all genes into nine groups labelled A to I. Genes in group C were upregulated in KD and downregulated in RE compared with WT (UP Group). Genes in group G were downregulated in KD and upregulated in RE compared with WT (DOWN Group). (B‐D) Scaled expression of genes in UP and DOWN groups (B), cell cycle (C), and spindle component (D). (E, F) Enrichment analysis of genes in the DOWN group using GO biological process terms (E) and KEGG pathways (F). The clusterProfiler package was used to perform the enrichment analysis

To determine the molecular mechanism of WDR62 in myocardial cell mitosis, we performed RNA‐seq analysis to identify differentially expressed genes among control (CON), KD, and RE groups (Figure 5). Genes most associated with *Wdr62* expression were defined as those with significantly different expression in KD that recovered to an extent (i.e., were more similar to the control) in RE. Using these criteria, 1883 genes were selected for enrichment analysis using Gene Ontology (GO) and Kyoto Encyclopedia of Genes and Genome (KEGG) (Figure 5A). Among these genes, 1065 downregulated key genes (DOWN group) were enriched in bioprocesses and pathways of cardiomyogenesis (Figure 5B–F), and 12 genes involved in cell cycle, spindle function, and cardiomyocyte proliferation‐related Hippo signaling (CORE genes) were validated with qRT‐PCR (Figure 5C–D, Supplementary Figure S7A). In addition, we found the expression of validated proliferation‐related genes *E2f2*, *Id1*, *Birc5*, *Cdc45*, *Mcm3*, *Chek1*, *Pttg1*, *Cdc25c*, and *Ccnb2* were significantly related to *Wdr62* through published microarray data from 136 heart‐related samples at different stages of development (Supplementary Figure S7B). However, upregulated genes (UP group) were not significantly enriched in pathways associated with cardiac development.

**FIGURE 6:**
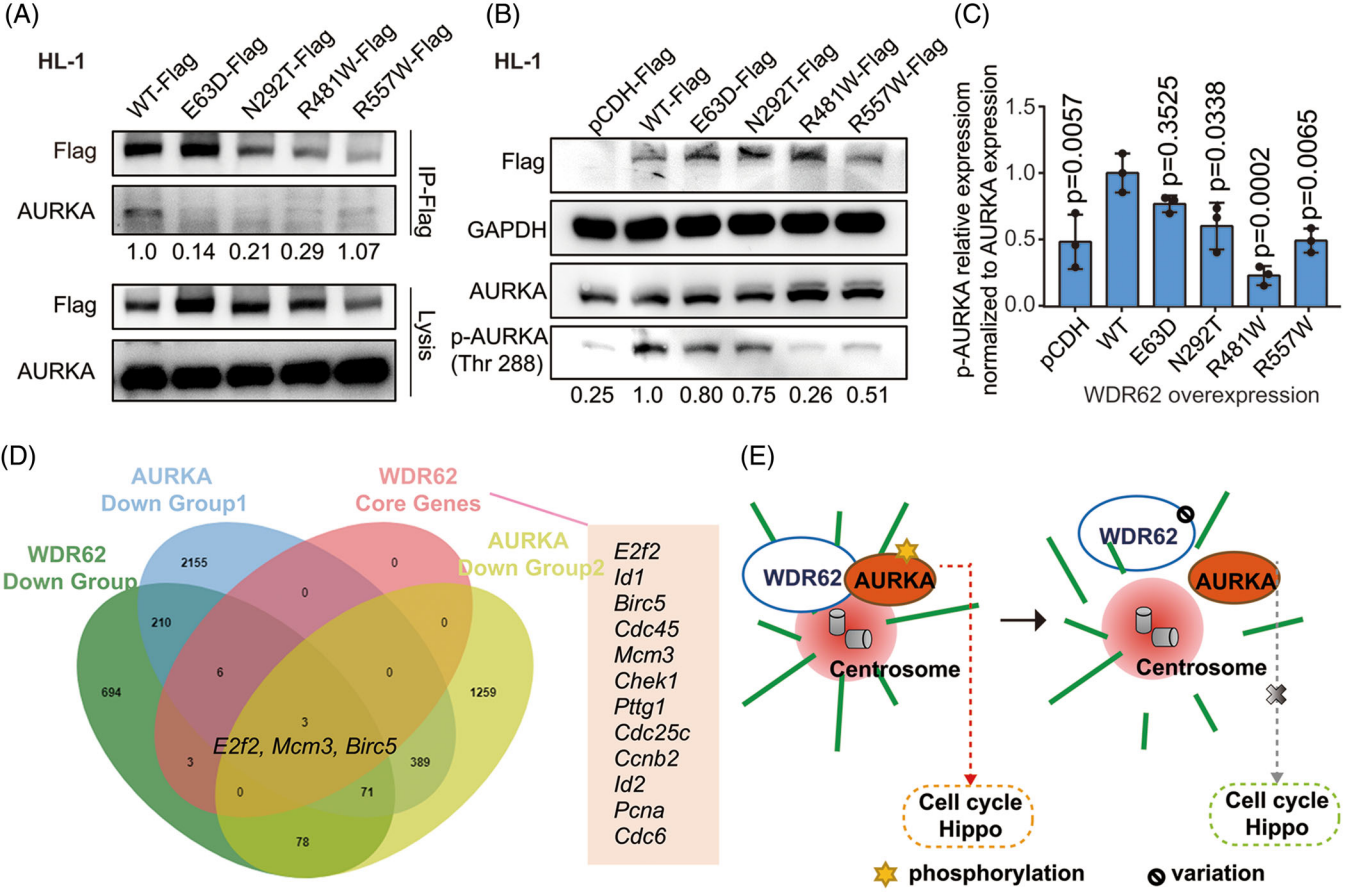
The effects of WDR62 variants on AURKA. (A) Co‐IP analysis of WDR62–AURKA interactions in HL‐1 cells using Flag antibodies, followed by probing with AURKA antibody. HL‐1 cells were transfected with expression plasmids of *WDR62*‐WT or one of four *WDR62* variants. Less AURKA was captured when four WDR62 variants were overexpressed. Representative images from four independent experiments are shown. The captured amount of AURKA in the representative images was normalized to that of Flag and the normalized values by greyscale scanning are indicated. (B) Effects of WDR62 variants on AURKA phosphorylation (p‐AURKA). The levels of p‐AURKA (Thr 288) in HL‐1 cells with *WDR62*‐WT overexpression were higher than that in cells overexpressing empty vector or four *WDR62* variants. Representative images from three independent experiments are shown. The captured amount of pAURKA in the representative images was normalized to that of AURKA and the normalized values by greyscale scanning are indicated. (C) Semi‐quantitative analysis of p‐AURKA levels relative to the expression levels of AURKA was performed by greyscale scanning of strips. The data are expressed as mean ± SD of three independent experiments. *p*‐values indicate comparison versus WT. (D) Venn diagrams showing overlap of downregulated genes in the DOWN group, CORE genes and two online transcriptome profiles of AURKA downregulation (online transcript GSE23541 and GSE57810). (E) Illustration showing the WDR62‐AURKA regulation of spindle assembly and cardiomyocyte proliferative pathways and the harmful effects of WDR62 variants

Co‐immunoprecipitation (Co‐IP) in HL‐1 and human embryonic kidney cell line HEK293T indicated that WDR62 could interact with key mitotic kinases AURKA. AURKA plays an essential role in spindle assembly and its deficiency results in mitotic arrest and disordered spindle formation.^35^ This interaction was weakened in some WDR62 variants (Figures 6A, Supplementary Figure S8A‐C), and their co‐location on spindle poles was changed by WDR62 variants, as determined through immunofluorescence (Supplementary Figure S8D). WDR62 variants could also reduce the activation of AURKA, as indicated by AURKA phosphorylation levels (Thr 288) (Figure 6B,C). Interestingly, by merging the CORE genes with downregulated genes caused by AURKA knockdown (from online transcript profiles GSE23541 and GSE57810), we found that both WDR62 and AURKA can regulate the expression of *E2f2, Mcm3*, and *Birc5* (Figure 6D). After treatment with MLN8237, an inhibitor of AURKA, the expression of *E2f2, Mcm3*, and *Birc5* decreased in HL‐1 cells (Supplementary Figure S8E). These data indicated that the interaction of WDR62 with AURKA has a potential role in proliferation regulation. Taken together, our results indicated that WDR62 may participate in the proliferation of cardiomyocytes by regulating the activities of AURKA to affect spindle assembly and related genes of cell cycle and Hippo pathways (Figure 6E).

## 4 DISCUSSION

CHD is the most common birth defect worldwide and has high heritability. Genetic etiology studies have been confounded by the existence of unknown CHD genes, high locus heterogeneity, low penetrance, and the lack of signatures for loss‐of‐function intolerance.^16^, ^36^ Large numbers of CHD‐related genes need to be further explored.^37^

In our study, because we focused on the identification of the novel susceptibility gene for CHD, all patients enrolled only had CHD. At first, we found the variants of nine genes were enriched in TOF individuals by sequencing and case‐control analysis. *JAG1* as a well‐studied susceptibility gene for TOF ranked first, which illustrated the validity of our cohort analysis to some extent.^32^, ^38^ Then, *WDR62* was screened out for further study because its degree of enrichment of variant enrichment in our cohort was the second highest and the association with CHD has not been reported. In zebrafish, the knockdown of *wdr62* caused impaired OFT rotation and TOF‐related defects. Unlike the human *WDR62‐*WT mRNA, the variant mRNAs were unable to rescue heart defects caused by *wdr62* knockdown, which indicated that the seven identified TOF variants were loss‐of‐function variants. Based on these results, *Wdr62* knockout mice models were used for further study. Corresponding to the abundant expression of WDR62 in OFT and RV of embryonic hearts, *Wdr62* homozygous global knockout mice exhibited a series of heart defects affecting OFT and RV. Although the phenotypes of TOF were observed, the most frequently occurring defect was VSD. VSD is a common phenotype in CHD, which could result from disturbances of both myocardial component (in‐ventricle and/or interventricular septum) developments and OFT separation.^39^, ^40^ Although both the incidence and severity of malformation were lower in HE mice than that in *Wdr62*‐null, the rate of heart defects of HE mice was significantly increased when compared with WT, corresponding to the heterozygous *WDR62* variants found in TOF patients. In addition, we also constructed a conditional knockout mice model with *Nkx2.5‐Cre* lines that deleted *Wdr62* in pharyngeal endoderm, myocardium, endocardium, and epicardium^41^ and only observed that very few presented mild VSD. It has been reported that interactions of the surrounding environment (neural crest and macrophage, for example) with cardiomyocyte are critical for appropriate proliferation and growth of the myocardium during cardiac development.^42^, ^43^, ^44^ This might explain why the phenotype of heart defects in *WDR62* conditional knockout mice was not obvious.

Based on the frequency and types of heart abnormalities observed in *Wdr62*‐null mice, a larger CHD cohort with corresponding subtypes was recruited. In total, our study detected the potentially risk variants of *WDR62* in about 6% of 1320 CHD patients, which is similar to or even higher than the variants of other CHD candidate genes.^45^, ^46^, ^47^ Additionally, as the CHD type frequently related with abnormal OFT and RV, VSD accounted for a higher proportion in our CHD samples with *WDR62* variants (8% of 718 patients).

WDR62 has been reported to be engaged in microcephaly, reproductive system diseases, and cancer in humans.^48^, ^49^, ^50^, ^51^ Correspondingly, the *Wdr62*‐null mice also showed microcephaly and all were infertile. Notably, distinct CHD phenotypes were observed in *Wdr62*‐null mice and the heart defects, rather than microcephaly, were the only deformities of which the ratio was significantly increased in HE mice compared with WT. Given that *WDR62* was first identified to be associated with microcephaly, clinical examinations and phenotypic recordings of carriers with *WDR62* variants were often focused on neurological, cognitive, facial, and behavioural aspects, and the information on the hearts were unavailable in previous studies.^50^, ^52^ As the diagnosis of CHD requires the help of specific examination methods such as echocardiography, whether these carriers had cardiac abnormalities was unknown. Additionally, if the carriers with *WDR62* variants in the previous study really did not present CHD phenotype, it also may be the following reasons: (1) the penetrance of *WDR62* variants varies in the heart and brain, which may be due to tissue‐specific mechanisms of dosage compensation^53^ and (2) *WDR62* may be a genetic modifier which contributes to CHD with other gene variants.^54^ Furthermore, another study reported two patients with primary amenorrhea carried deleterious *WDR62* heterozygous mutations and no one had microcephaly.^49^

Furthermore, microcephaly, growth retardation, microphthalmia, and embryonic lethality observed in our *Wdr62*‐null mice were largely consistent with another homozygous *Wdr62* mutant model (WDR62^stop/stop^), which were also constructed with CRISPR/Cas9 editing technology.^55^ However, whether infertility and heart defects were observed in this model was not mentioned. The discrepancy may be due to different construction strategies: The WDR62^stop/stop^ lost 1 base pair in exon 2 resulting in pre‐termination codon; in our model, core promoter and exon 1 were deleted, which could lead to a more thorough knockout. Another reason may be that the authors did not pay much attention to the phenotype of some internal organs such as the heart. Variants in genes known to cause isolated CHD can also result in syndromic CHD.^53^ Considering that all patients enrolled in our study only had CHD, whether our *WDR62* variants are associated with microcephaly and reproductive disorders should be clarified in further research.

Abnormal cell cycle and spindle assembly related to proliferation of cardiomyocytes were observed in animal models and cell lines with WDR62 deficiency. At E9.5 in mice, the heart begins significant proliferation and growth that leads to septation.^42^ WDR62 had the highest expression in the heart on E9.5, and the expression dynamics in the heart during development were similar to these of myocardial proliferative activity.^56^ Disturbance of proliferation results in various structural defects including abnormal alignment of the aorta, VSD, and cardiomyopathy.^57^, ^58^, ^59^ These abnormalities were similar to those observed in our CHD patients with *WDR62* variants and *Wdr62*‐null mice. A previous publication showed *Pcnt* knockout mice presenting muscular VSD may be related to malalignment of the spindle apparatus in cells during the process of forming ventricular septum.^60^ Our study indicated not only a role for *WDR62* variants in causing VSD from mitotic spindle defects, but in other CHD defects such as TOF. Notably, although the abnormal spindle assembly and mitosis arrest caused by WDR62 deficiency can also destroy self‐renewal and fate specification of neural stem/progenitor cells,^48^ the control of brain growth by WDR62 predominantly depends upon its glial lineage function. And the decreased number of neural stem cell (neuroblast) caused by WDR62 deficiency might be rescued by the compensatory proliferation of progeny cells and the antagonistic effect of other proteins.^61^ Coupled with the regeneration ability of cells still existing in adult brain rather than heart, the heart seems to be more sensitive to the proliferative defects caused by WDR62 haploinsufficiency than brain. These may be the reasons that a certain proportion of HE mice only exhibited cardiac malformations rather than microcephaly.

WDR62 has been reported to regulate the orientation and organization of spindle poles by interacting with AURKA.^61^ AURKA knockout mice exhibited embryonic death with severe cell proliferated failure due to mitotic arrest (significant increase in the percentage of PHH3+ cells) and abnormal spindle formation.^35^, ^62^ This is consistent with our results in cardiomyocytes, where *WDR62* variants could impair the interactions with and activities of AURKA, which may connect with abnormal spindle assembly. AURKA could affect the expression of genes related to cell proliferation through multiple signalling pathways (AKT, MAPK, etc.).^61^, ^63^ Our RNA‐seq data also indicated that WDR62 has a potential role in regulating genes associated with heart development and myocyte proliferation. Interestingly, by joint analysis of multiple transcript profiles, some of the downstream genes involved in myocyte proliferation of WDR62 and AURKA were shown to overlap, such as *E2f2*, *Mcm3*, and *Birc5*. E2F2 is a key transcription factor regulating cell cycle genes in cardiomyocytes^64^; MCM3 is involved in the initiation of replication and is regulated by E2F^65^, ^66^; BIRC5 participates in centre spindle organization and is the target gene of Hippo signaling.^67^, ^68^ Corresponding to these, by using AURKA inhibitor MLN8237 in HL‐1, the expression of these three genes was also reduced. These data indicate the potential role of WDR62–AURKA interaction in cell proliferation by regulating spindle assembly and the expression of downstream genes involved in cell cycle and Hippo signalling.

In conclusion, our study provided extensive evidence for the association of WDR62 and cardiac development. We identified *WDR62* as a novel susceptibility gene of CHD with high variant frequency, especially in VSD. WDR62 was shown to participate in cardiac development by affecting spindle assembly and cell cycle in cardiomyocytes. The recruitment of CHD pedigrees for co‐segregation analysis and more matched controls for association analysis will help to confirm the genetic contribution of *WDR62* to CHD.

## CONFLICT OF INTEREST

The authors declare no conflict of interest.

## Supporting information



Supporting Information

## Data Availability

The data that support the findings of this study are available on request from the corresponding author.
