# Supplementary material for: WDR62 variants contribute to congenital heart disease by inhibiting cardiomyocyte proliferation
Source: Clin Transl Med. 2022 Jul 8;12(7):e941. doi: 10.1002/ctm2.941 (PMC9270576; doi:10.1002/ctm2.941)
Supplement: Supplementary file 1 — Supporting Information [file CTM2-12-e941-s001.pdf]

SUPPLEMENTAL INFORMATION

SUPPLEMENTAL METHODS

Sanger sequencing of all exons of *WDR62*

A total of 200 unrelated TOF patients were recruited for Sanger sequencing. The coding exons of *WDR62* (NM_001083961.2) were amplified using custom intronic primers and standard PCR protocols (primer sequences are available on request) combined with Sanger sequencing. Amplicons were sequenced using an ABI Prism 3730XL Genetic Analyzer (Applied Biosystems) according to the manufacturer's instructions.

Zebrafish lines and feeding

For all zebrafish experiments, wild type zebrafish embryos of the Tu and transgenic cmlc2: GFP (cardiac myosin light chain 2: eGFP reporter) lines [Tg (cmcl2: GFP)] were used. The Tg (cmcl2: GFP) line was established by Tol2 system. The primers to get the promoter of zebrafish *cmlc2* were as follows: cmlc2-F, 5'-CCCTTTCTGCAGCTTTGTTT-3'; cmlc2-R, 5'-GCCTCTTTGAGCAGTTTTCC-3'.

Zebrafish were reared under standard aquaculture conditions at 28.5 °C on a 14/10 hour light/dark cycle. Embryos were collected after group mating and kept in embryo medium (17 mM NaCl, 0.2 mM KCl, 0.18 mM $Ca(NO_3)_2$, 0.12 mM $MgSO_4$, 1.5 mM HEPES buffer pH 7.1-7.3 and 0.6 μM methylene blue).

Whole-mount in situ hybridization

The zebrafish *wdr62* (*zwdr62*) cDNA sequence was downloaded from ensemble network and

the Genscan ID was ENSDARG00000060312. The *zwdr62* probes used in the experiment were from +2037 to +2964, which were amplified using specific primers (*zwdr62*-ISH-F: 5'-ATCTGTTGGCTTCAGCGAGT-3'; *zwdr62*-ISH-R: 5'-GGTTTGGAGCACCACAGAAT-3') and was cloned into the pGEM®-T Easy Vector (Promega). Digoxigenin (Dig)-labeled probes were synthesized by in vitro transcript reaction using dig-RNA labeling kit (Roche) following the manufacturers' instructions. Embryos from 12, 24, 48 and 72 hpf were fixed in 4% paraformaldehyde (PFA) overnight. Hybridization was performed as described previously.[1] The embryos were imaged using a Leica M205C microscope.

Cardiac phenotype observation in zebrafish

Embryos at 48, 72 and 96 hpf were collected for phenotype analysis. The embryos were fixed in methylcellulose and imaged using Leica M205C microscope. The images of 72 hpf hearts (GFP-labeled) were got by Lecia SP8 confocal microscope. The heart sections of zebrafish were used to H&E (hematoxylin-eosin) staining, and the pictures were taken by Lecia DM750. The counting of ventricle cardiomyocytes was performed as described previously.[2] DAPI and GFP double positive cells indicated as cardiomyocytes.

RNA preparation and quantitative real-time PCR (qPCR)

The total RNA was extracted using the TRIzol reagent (Invitrogen) and converted to cDNA using the PrimeScript RT Reagent Kit (Takara Bio). The qPCR reactions were performed with SYBR Premix Ex Taq (Takara) using the Roche 480 plus system (Roche). Primer sequences will be available on request.

Mice breeding and the construction of *Wdr62* knockout mice

All mouse lines were maintained in a C57BL/6J genetic background. The *Wdr62*-floxed

(*Wdr62*$^{fl/+}$) mice was generated using CRISPR-Cas9 technology at Beijing Biocytogen Co. Ltd. The loxp sites were inserted at both sides of exon 1 via homologous recombination. *CMV-Cre* transgenic mice were from Beijing Biocytogen Co.Ltd. *Nkx2.5-Cre* mice were from Jiangsu GemPharmatech Co.Ltd. The heterozygous *Wdr62* knockout mice were obtained by crossing *Wdr62*$^{fl/+}$ with *CMV-Cre* and then bred to obtain homozygous knockout mice (*Wdr62*-null). *Wdr62* $^{fl/fl}$ mice were bred with *Nkx2.5-Cre* mice to generate mice with deletion of *Wdr62* from the myocardium. Primers for genotyping Cre alleles: Cre-F, 5’-CAGAACCTGAAGATGTTCGCGAT-3’, Cre-R, 5’-CCTGATCCTGGCAATTTCGGC-3’, a PCR product of 347 bp. Primers for genotyping *Wdr62* floxed allele: 5’loxP-F, 5’-CAGCAAACTCAGCTCAACTCTGCAA-3’, 5’loxP-R, 5’-CTGGCTGTCCTGTGAGCATGTTGTA-3’. The amplified floxed allele was 434 bp, whereas the wild-type allele was 348 bp. Primers for verifying the occurrence of *Wdr62* knockout: 5’loxP-F, 5’-CAGCAAACTCAGCTCAACTCTGCAA-3’, 3’loxP-R, 5’-GGGAATGTTAACCAAATAGGGGCCT-3’, a PCR product of 271 bp. Embryos were dated taking the morning of the vaginal plug as embryonic day (E) 0.5.

Cardiac phenotype observation in mice

The pregnant mice were anaesthetized by isoflurane inhalation and euthanized by rapid cervical dislocation. Embryos of specific embryonic age were collected and photographed in phosphate-buffered saline (PBS) using a Leica M205C microscope. The ventricular area, and the OFT and RV length of mouse hearts were measured using Image J.

H&E staining and immunohistochemistry of paraffin section

The embryos were dehydrated in gradient concentration ethylalcohol and clarified in gradient

concentration xylene, and then embedded in paraffin. The paraffin sections were baked in 56 °C overnight. The section were dewaxed in xylene, gradient dehydrated in ethylalcohol and dip-dye in hematoxylin and eosin. For immunohistochemical analysis, sections underwent heat-induced antigen retrieval after deparaffinating and rehydration. Immunostaining was performed using the VECTASTAIN® Elite® ABC-HRP Kit (Vector Laboratories). All immunostained sections were observed by at least two independent pathologists in a blind manner.

Tissue immunofluorescence

The fresh hearts were embedded in OCT and saved in -80 °C before cryostat sectioning. After sectioning, the heart tissues were fixed in cold acetone and then were equilibrated in room temperature about 15 min. Primary antibodies were incubated overnight in the blocking buffer. Appropriate Alexa Fluor dye–conjugated secondary antibodies (Invitrogen) were incubated for 1 h along with DAPI for nuclei staining. Primary antibodies used on tissue sections were as follows: mouse anti-TNNT2 (MAB1874, R&D Systems); rabbit anti-WDR62 (A301-560A, Bethyl Laboratories); mouse anti-β-Tubulin (T4026, Sigma-Aldrich).

Multiplex PCR-targeting sequencing

Peripheral blood samples from 1083 patients were obtained. The patients include 234 TOF, 718 VSD, 55 PA+VSD, 19 DORV+PS and 57 RVD. Multiplex PCR-Targeting Sequencing was applied to sequence all exons, splice site (± 20bp of adjacent intronic sequences) and regulatory regions (5,000 bp upstream of the transcription start site, 300 bp downstream of the transcriptional termination site) of *WDR62*. Primes design covered a theoretical 99.9% of the complete target region. Pooled samples were sequenced on HiSeq 2500 (Illumina) platform.

The BWA software, SAM tools, GATK and dbSNP Build 150 were used to map sequence reads to the GRCh37, calculate read quality and call single-nucleotide variants. For variant filtration, allelic frequencies were compared to the 1000G, the Exome Aggregation Consortium (ExAC) and the GnomAD dataset. We prioritized variants with MAF< 1%. Additionally, synonymous variants and missense variants with the CADD Phred score≤ 20 or tolerated/benign status by SIFT and Polyphen 2 were excluded.

Validation of the new variants

The *WDR62* variants were validated by Sanger sequencing. PCR products were sequenced with an ABI 3730 Genetic Analyzer using their respective forward or reverse primers. The chromatograms were analyzed using the Mutation Surveyor DNA Variant Analysis Software (SoftGenetics).

Analysis of WDR62 variants through ACMG-AMP guidelines[3]

For each screened variant, we calculated OR for case-control associations and MAF in population databases including the 1000G (1000G_2015aug), the GnomAD, ESP6500SI-V2 and ExAC. The variant pathogenicity was evaluated by the site conservation, splice consensus and deleterious effects on encoded amino acids, regulatory DNA elements and microRNA binding.

(1) *MAF of WDR62 variants (**Supplementary Table 6**)*

The MAF for each identified variant in total or East Asian-matched population was extracted by 1000G_2015aug containing genomes of 2504 persons, the ESP6500SI-V2 containing exomes of 6503 persons, the ExAC containing exomes of 69796 persons, and the GnomAD which contain 125,748 exome sequences (GnomAD_exome) and 15708 whole-genome

sequences (GnomAD_genome). Variants with MAF≤ 0.1% were considered to be rare variants by the "PM2" evidence of ACMG-AMP guidelines.

(2) *Algorithms predicting pathogenicity of WDR62 variants*

For missense variants, algorithms evaluating the conservation of amino acid and the influence on protein function included SIFT, PolyPhen2-DVIV and HVAR, CADD, Mutation Taster and Mutation Assessor as found in ANNOVAR (**Supplementary Table 7**).

The evolutionary conservation of DNA sequence where *WDR62* variants occurred was analyzed by algorithms including PhyloP,[4] GERP++RS[5] and PhastCons-100 way[6] as found in Alamut Visual version 2.11 (Interactive Biosoftware). An allele conserved across various species maybe produce harmful effects by variations (**Supplementary Table 8**).

To analyze the effect of variants in the introns on splicing consensus, algorithms of SpliceSiteFinder,[7] MaxEntScan,[8] GeneSplicer[9] and NNSPLICE[10] were used to evaluate the changing of splicing signals, and the ESEFinder[11] and RESCUE-ESE[12] were applied to predict the alteration of exonic splicing enhancers sites (ESE) (**Supplementary Table 10**).

The variants located in the regulatory regions might affect the expression level of WDR62. The effect of these variants on regulatory DNA elements (DNase hypersensitivity regions, binding sites of transcription factors and promoter regions) were predicted by RegulomeDB v2.0.[13] Variants in Ranking 1 or 2 with Probability> 0.500 were assumed to affect the function of regulatory DNA elements (**Supplementary Table 11**).

Multiple previous studies have reported that variants in 3' UTR can affect mRNA-miRNA interaction by disrupting miRNA recognition element, which could finally alter gene expression and cause diseases.[14, 15] So we also studied whether the *WDR62* variants at 3'UTR

could affect microRNA binding. The prediction of the variant c.*36G>C were performed through Alamut Visual which was based on miRanda and microRNA.org (**Supplementary Table 12**).

The variant having impact on site conservation/splicing/encoded amino acids function/regulatory DNA elements/microRNA binding was predicted be harmful according to "PP3" evidence of ACMG-AMP guidelines.

(3) *Case-Control Association Studies (**Supplementary Table 13**)*

The frequency of *WDR62* variants between the population with CHD and the controls from East Asian groups of GnomAD database was compared using two-sided Fisher's exact test. OR= 1 indicated that the variants had no effect on the risk of the disease. OR> 1 indicated the variants increased the risk of the disease. In our study, variants with OR> 5 and *p*-value< 0.050 were considered to be associated with disease significantly according to the "PS4" evidence of ACMG-AMP guidelines.

The pathogenic classification of all *WDR62* was made according to rules for combining criteria set by ACMG-AMP workgroup. The variants were classed as "Established risk allele", "likely risk allele" or "Uncertain risk allele".

WDR62 expression plasmids, cell transfection, lentivirus packaging and infection

HL-1 and HEK293T cells were cultured in Dulbecco's Modified Eagle's Medium supplemented with 10% fetal bovine serum (FBS) and 1% Pen-Strep antibiotics at 37 °C and 5% CO_2.

WDR62-WT was tagged at the N and C terminus with Flag-tag and then was cloned into the Not I-HF and Nhe I sites of the mammalian expression vector pCDH-CMV-MCS-EF1-copGFP

(WDR62-WT-Flag). The 7 variants plasmids were created respectively (pCDH-E63D-Flag, pCDH-N292T-Flag, pCDH-R481W-Flag, pCDH-R557W-Flag, pCDH-C599S-Flag, pCDH-C806W-Flag and pCDH-R1096H-Flag) using the KOD-Plus Mutagenesis Kit. All the expression plasmids were fully sequenced. The WDR62-WT-Flag or *WDR62* variant constructs were transiently overexpressed using Viafect transfection agent (Promega).

Stable gene overexpression constructs were established through cloning Flag-tagged WDR62-WT or variants (c.G189T, c.A875C, c.C1441T and c.C1669T) into the pCDH-CMV-MCS-EF1a-GFP+BSD (YouBio).

WDR62 were knocked down by CRISPR-Cas9. The *Wdr62* CRISPR gRNA were designed using the web tool developed by Feng Zhang group at MIT and subcloned between the two BsmBI sites of the lentiCRISPRv2 vector from Dr. Feng Zhang's laboratory. The gRNA target sequence was 5'-TTGGCTTCAGGCGACCGCAG-3'.

For the construction of stable cell lines, the pPACKH1™ HIV Lentivector Packaging Kit was used to package pseudoviral particles of the gRNA or gene expression constructs in HEK293T according to the manufacturer's instructions (LV500A−1, Systems Biosciences). The virus containing supernatant was collected, filtered (with 0.45 µm syringe filters) and infected the cells in complete media containing 5 µg/mL polybrene (Sigma-Aldrich). Puromycin dihydrochloride (Amresco) or Blasticidin S (Amresco) were used to screen the stable transfected cells after 48 h of infection.

Preparation of CON, KD and RE cell lines

HL-1 cells were stable transfected with pCDH-CMV-MCS-EF1a-GFP+BSD and lentiCRISPRv2 to establish CON cell line. For KD cells, WDR62 were knocked down in HL-

1 by CRISPR-Cas9. RE cell line was built by stable transfecting WDR62-WT-Flag in KD cells.

Cell immunofluorescence

The HL-1 cells were harvested 48 h after transfection. Then the cells were fixed with 4% PFA for 10 min, permeabilized with 0.2% Triton X-100 for 15 min and blocked with 3% BSA for 40 min in room temperature. Staining was carried out using the following antibodies: mouse anti-Flag (M20008S, Abmart), rabbit anti-WDR62 (A301-560A, Bethyl Laboratories), mouse anti-β-Tubulin (T4026, Sigma-Aldrich), rabbit anti-γ-Tubulin (ab179503, Abcam), rabbit anti-AURKA (14475, CST) and then detected with the Alexa Fluor dye–conjugated secondary antibodies (Invitrogen). Glass bottom plates were coated with Diamond anti-fade agent (Life technologies) and examined using Lecia SP8 confocal microscope.

Analysis of cell cycle and spindles

Cell samples for cell cycle analysis were prepared using The CycleTEST™ PLUS DNA Reagent Kit (BD Biosciences), the HL-1 single cell suspensions of CON, KD and RE were analyzed by flow cytometry (BD Biosciences).

For observations of spindle poles, cells were fixed for immunofluorescence with rabbit anti-γ-Tubulin (ab179503, Abcam) and the secondary Alexa Fluor 488 goat anti-rabbit IgG (Invitrogen). Transfected cells overexpressing WDR62-WT or variants were indicated by mouse anti-Flag (M20008S, Abmart) and the secondary Alexa Fluor 555 goat anti-mouse IgG (Invitrogen). Only one spindle pole or more than two spindle poles presented in one mitotic cell were identified to be the cell with abnormal spindles. Percentage of abnormal spindles (%) = the number of transfected cells with abnormal spindles/ the number of all mitotic transfected cells * 100%. More than five different views of each group were quantified.

RNA-seq and analysis

Three groups (CON, KD and RE) of HL-1 cells were obtained and each group included four replicates (10^7 cells each replicate). The RNA sequencing was carried on by the HaploX Biotechnology, Shenzhen, China. The FASTQ data of sequencing reads were trimmed using the program trim_galore (version 0.6.4) with parameters, "--paired --illumina", to remove low quality reads and adapter reads. Consecutively, we mapped the trimmed reads to the mouse genome reference (UCSC mm10) using the software TopHat (version 2.1.1) with default parameters. The program Cufflinks (version 2.2.1) with default parameters was used to assign the mapped reads to mouse transcripts (UCSC mm10) for identification of the gene expression abundance, represented by FPKM (Fragments Per Kilobase of transcript per Million mapped reads). The program cuffdiff in the Cufflinks suite with default parameters was used to calculate the fold-change and *p*-value of genes for comparison between samples. The enrichment analyses of GO and KEGG were performed using the ClusterProfiler R-package (version 3.18.0).

Correlation analysis

The expression levels of *Wdr62*, *E2f2, Id1, Id2, Birc5, Cdc45, Mcm3, Chek1, Pttg1, Cdc25c* and *Ccnb2* were obtained from microarray dataset (GSE1479, GSE5298, GSE30593, GSE40260, GSE14906, GSE32078, GSE43197, GSE9124 and GSE70254). The expression-correlations between *Wdr62* and the validated gene were analyzed by Liner regression with GraphPad Prism Software (version 7). Pearson correlation or Spearman test were used.

Western blotting

Tissue and cell were lysed in cold RIPA buffer (Calbiochem/EMD Millipore) with protease

inhibitor cocktail (Roche) and phenylmethanesulfonyl fluoride solution (PMSF) (Sigma-Aldrich). The protein lysates were separated by SDS–PAGE and were transferred onto nitrocellulose membranes, blocked in 8% milk and incubated with the primary antibodies. Primary Antibodies were as follows: rabbit anti-WDR62 (ab154044, Abcam), rabbit anti-WDR62 (A301-560A, Bethyl Laboratories); mouse anti-β-tubulin (T4026, Sigma-Aldrich), GAPDH (60004-1-Ig, Proteintech); mouse anti-Flag antibody (M20008S, Abmart), AURKA (ab108353, Abcam), phospho-Aurora A (Thr288) (2914, CST). The membranes were then washed and incubated with secondary HRP-conjugated antibodies (Proteintech). SuperSignal™ West Pico PLUS Chemiluminescent Substrate (Thermo Fisher) was used to visualize proteins on X-ray film. Uncropped blots were shown in Source Data.

Protein-protein interaction identification

Protein-protein interactions were verified in HL-1 cells after overexpression of WDR62-WT or WDR62 variants. Cell lysates were obtained in RIPA containing 1 mM PMSF and protease inhibitor cocktail (Roche). Immunoprecipitation was performed by anti-Flag affinity gel (Biotool). SDS-PAGE was performed to resolve the eluates. The interaction between WDR62 and AURKA was detected by Co–IP and western blot with rabbit anti−AURKA (ab108353, Abcam) and anti−Flag (M20008S, Abmart).

SUPPLEMENTAL TABLES (S1-S18)

Table S1. Statistics of genes with probable pathgenic variants detected by whole-exome sequencing in TOF cases versus normal controls

Genes[†]	The number of TOF cases with	The number of controls with probable	p value[‡]	Adjust p value[§]	Odds ratio (OR)	OR_ lowest	OR_ highest

	probable pathgenic variants (n= 37 in total)	pathgenic variants (n= 207 in total)					
JAG1	5	1	0.0003	0.0751	31.56	3.38	1522.24
WDR62	4	1	0.0020	0.0915	24.52	2.34	1231.61
KNDC1	6	5	0.0021	0.0915	7.75	1.85	34.20
DRD3	3	0	0.0032	0.0915	Inf	2.40	Inf
OGFOD2	3	0	0.0032	0.0915	Inf	2.40	Inf
PHF12	3	0	0.0032	0.0915	Inf	2.40	Inf
SRPX2	3	0	0.0032	0.0915	Inf	2.40	Inf
TSKS	3	0	0.0032	0.0915	Inf	2.40	Inf
MEGF8	5	4	0.0049	0.1019	7.86	1.60	41.81
AGAP1	4	2	0.0054	0.1019	12.27	1.68	140.85
EMILIN2	4	2	0.0054	0.1019	12.27	1.68	140.85
FZD10	4	2	0.0054	0.1019	12.27	1.68	140.85
APC2	6	7	0.0062	0.1083	5.50	1.43	20.55
FSCN2	4	3	0.0111	0.1138	8.16	1.32	58.29
MEF2A	4	3	0.0111	0.1138	8.16	1.32	58.29
AHNAK2	3	1	0.0115	0.1138	17.90	1.39	957.13
ANO8	3	1	0.0115	0.1138	17.90	1.39	957.13
C10orf90	3	1	0.0115	0.1138	17.90	1.39	957.13
NHLRC3	3	1	0.0115	0.1138	17.90	1.39	957.13
SARM1	3	1	0.0115	0.1138	17.90	1.39	957.13
SDPR	3	1	0.0115	0.1138	17.90	1.39	957.13
SLC6A5	3	1	0.0115	0.1138	17.90	1.39	957.13
TSC22D4	3	1	0.0115	0.1138	17.90	1.39	957.13
FARP2	6	9	0.0142	0.1332	4.24	1.16	14.46
LTK	4	4	0.0199	0.1332	6.11	1.08	34.50
MAST4	4	4	0.0199	0.1332	6.11	1.08	34.50
MYO7B	4	4	0.0199	0.1332	6.11	1.08	34.50
MYO9B	4	4	0.0199	0.1332	6.11	1.08	34.50
PCNXL3	4	4	0.0199	0.1332	6.11	1.08	34.50
SLC12A4	4	4	0.0199	0.1332	6.11	1.08	34.50
LRBA	5	7	0.0215	0.1332	4.45	1.05	17.43
MAGEF1	5	7	0.0215	0.1332	4.45	1.05	17.43
UNC13D	5	7	0.0215	0.1332	4.45	1.05	17.43
AP5Z1	3	2	0.0257	0.1332	8.95	0.99	110.89
CAMSAP2	3	2	0.0257	0.1332	8.95	0.99	110.89
CCDC64B	3	2	0.0257	0.1332	8.95	0.99	110.89
ILDR1	3	2	0.0257	0.1332	8.95	0.99	110.89
NUP133	3	2	0.0257	0.1332	8.95	0.99	110.89
OR10A4	3	2	0.0257	0.1332	8.95	0.99	110.89

PARD3B	3	2	0.0257	0.1332	8.95	0.99	110.89
SNTB1	3	2	0.0257	0.1332	8.95	0.99	110.89
SORCS1	3	2	0.0257	0.1332	8.95	0.99	110.89
TNRC18	3	2	0.0257	0.1332	8.95	0.99	110.89
ULK4	3	2	0.0257	0.1332	8.95	0.99	110.89
MYH15	5	8	0.0310	0.1398	3.88	0.94	14.44
NIN	5	8	0.0310	0.1398	3.88	0.94	14.44
FOCAD	4	5	0.0319	0.1398	4.87	0.92	23.95
JAG2	4	5	0.0319	0.1398	4.87	0.92	23.95
KLHDC4	4	5	0.0319	0.1398	4.87	0.92	23.95
MYH7B	4	5	0.0319	0.1398	4.87	0.92	23.95
TIMELESS	4	5	0.0319	0.1398	4.87	0.92	23.95
UNC45B	4	5	0.0319	0.1398	4.87	0.92	23.95
ATP2B2	3	3	0.0460	0.1608	5.96	0.77	46.34
HARS	3	3	0.0460	0.1608	5.96	0.77	46.34
KY	3	3	0.0460	0.1608	5.96	0.77	46.34
LOC93432	3	3	0.0460	0.1608	5.96	0.77	46.34
NOS1	3	3	0.0460	0.1608	5.96	0.77	46.34
NRIP2	3	3	0.0460	0.1608	5.96	0.77	46.34
SCIN	3	3	0.0460	0.1608	5.96	0.77	46.34
SLC22A25	3	3	0.0460	0.1608	5.96	0.77	46.34
SSH2	3	3	0.0460	0.1608	5.96	0.77	46.34
SYTL3	3	3	0.0460	0.1608	5.96	0.77	46.34
ZFAND4	3	3	0.0460	0.1608	5.96	0.77	46.34
ZNF236	3	3	0.0460	0.1608	5.96	0.77	46.34
SYNE1	3	47	0.0469	0.1608	0.30	0.06	1.03
SOGA1	4	6	0.0474	0.1608	4.05	0.80	18.13
WNK1	4	6	0.0474	0.1608	4.05	0.80	18.13
TRIOBP	6	13	0.0480	0.1608	2.89	0.84	8.91
KNTC1	5	10	0.0572	0.1850	3.07	0.77	10.68
RELN	5	10	0.0572	0.1850	3.07	0.77	10.68
GSE1	4	7	0.0664	0.1850	3.46	0.70	14.50
NWD1	4	7	0.0664	0.1850	3.46	0.70	14.50
ACPP	3	4	0.0722	0.1850	4.46	0.63	27.61
AGBL3	3	4	0.0722	0.1850	4.46	0.63	27.61
ALPK1	3	4	0.0722	0.1850	4.46	0.63	27.61
AMOTL2	3	4	0.0722	0.1850	4.46	0.63	27.61
CAPN3	3	4	0.0722	0.1850	4.46	0.63	27.61
CCDC150	3	4	0.0722	0.1850	4.46	0.63	27.61
GOLGA5	3	4	0.0722	0.1850	4.46	0.63	27.61
GP6	3	4	0.0722	0.1850	4.46	0.63	27.61
MYO19	3	4	0.0722	0.1850	4.46	0.63	27.61
NOL8	3	4	0.0722	0.1850	4.46	0.63	27.61
OLFML2A	3	4	0.0722	0.1850	4.46	0.63	27.61

PAPLN	3	4	0.0722	0.1850	4.46	0.63	27.61
PDCD11	3	4	0.0722	0.1850	4.46	0.63	27.61
PLEKHG5	3	4	0.0722	0.1850	4.46	0.63	27.61
PTPN14	3	4	0.0722	0.1850	4.46	0.63	27.61
TEKT3	3	4	0.0722	0.1850	4.46	0.63	27.61
TNS3	3	4	0.0722	0.1850	4.46	0.63	27.61
OTOGL	5	11	0.0741	0.1877	2.78	0.71	9.42
TTN	24	101	0.0759	0.1903	1.95	0.90	4.41
AIM1	4	8	0.0889	0.2179	3.01	0.63	12.04
KMT2D	4	8	0.0889	0.2179	3.01	0.63	12.04
ARHGEF11	3	5	0.1036	0.2250	3.56	0.53	19.26
CEP152	3	5	0.1036	0.2250	3.56	0.53	19.26
DFNB31	3	5	0.1036	0.2250	3.56	0.53	19.26
EEF1D	3	5	0.1036	0.2250	3.56	0.53	19.26
FCHSD1	3	5	0.1036	0.2250	3.56	0.53	19.26
MUT	3	5	0.1036	0.2250	3.56	0.53	19.26
NDST2	3	5	0.1036	0.2250	3.56	0.53	19.26
PRPF40B	3	5	0.1036	0.2250	3.56	0.53	19.26
SAMD9	3	5	0.1036	0.2250	3.56	0.53	19.26
SLC5A9	3	5	0.1036	0.2250	3.56	0.53	19.26
TLN2	3	5	0.1036	0.2250	3.56	0.53	19.26
WDFY3	3	5	0.1036	0.2250	3.56	0.53	19.26
ASPM	4	9	0.1146	0.2433	2.67	0.57	10.25
MYO5C	4	9	0.1146	0.2433	2.67	0.57	10.25
DNHD1	6	16	0.1153	0.2433	2.31	0.69	6.84
ALS2CL	3	6	0.1395	0.2606	2.95	0.46	14.63
CACNA1A	3	6	0.1395	0.2606	2.95	0.46	14.63
CFAP61	3	6	0.1395	0.2606	2.95	0.46	14.63
CROT	3	6	0.1395	0.2606	2.95	0.46	14.63
EGFLAM	3	6	0.1395	0.2606	2.95	0.46	14.63
FBN2	3	6	0.1395	0.2606	2.95	0.46	14.63
KIAA2026	3	6	0.1395	0.2606	2.95	0.46	14.63
KIF19	3	6	0.1395	0.2606	2.95	0.46	14.63
KMT2A	3	6	0.1395	0.2606	2.95	0.46	14.63
KMT2B	3	6	0.1395	0.2606	2.95	0.46	14.63
MADD	3	6	0.1395	0.2606	2.95	0.46	14.63
MCM3AP	3	6	0.1395	0.2606	2.95	0.46	14.63
MMACHC	3	6	0.1395	0.2606	2.95	0.46	14.63
STAB1	3	6	0.1395	0.2606	2.95	0.46	14.63
CSMD3	5	13	0.1618	0.2893	2.33	0.61	7.59
DOCK6	5	13	0.1618	0.2893	2.33	0.61	7.59
KIF26B	5	13	0.1618	0.2893	2.33	0.61	7.59
ADAMTS9	3	7	0.1789	0.2893	2.52	0.40	11.73
ALK	3	7	0.1789	0.2893	2.52	0.40	11.73

BRWD1	3	7	0.1789	0.2893	2.52	0.40	11.73
DHX34	3	7	0.1789	0.2893	2.52	0.40	11.73
DNAH7	3	7	0.1789	0.2893	2.52	0.40	11.73
HEPHL1	3	7	0.1789	0.2893	2.52	0.40	11.73
HK3	3	7	0.1789	0.2893	2.52	0.40	11.73
KIAA1109	3	7	0.1789	0.2893	2.52	0.40	11.73
KIF26A	3	7	0.1789	0.2893	2.52	0.40	11.73
LIMK2	3	7	0.1789	0.2893	2.52	0.40	11.73
PTPRU	3	7	0.1789	0.2893	2.52	0.40	11.73
RBP3	3	7	0.1789	0.2893	2.52	0.40	11.73
SHC2	3	7	0.1789	0.2893	2.52	0.40	11.73
TAS1R3	3	7	0.1789	0.2893	2.52	0.40	11.73
TNK2	3	7	0.1789	0.2893	2.52	0.40	11.73
UGGT2	3	7	0.1789	0.2893	2.52	0.40	11.73
ABCB5	3	8	0.2211	0.3361	2.20	0.36	9.75
ADAMTSL1	3	8	0.2211	0.3361	2.20	0.36	9.75
DNAH6	3	8	0.2211	0.3361	2.20	0.36	9.75
IQGAP3	3	8	0.2211	0.3361	2.20	0.36	9.75
PCDH15	3	8	0.2211	0.3361	2.20	0.36	9.75
PIWIL3	3	8	0.2211	0.3361	2.20	0.36	9.75
PLEKHD1	3	8	0.2211	0.3361	2.20	0.36	9.75
PLXNA4	3	8	0.2211	0.3361	2.20	0.36	9.75
TRIO	3	8	0.2211	0.3361	2.20	0.36	9.75
PIEZO1	4	11	0.2538	0.3807	2.16	0.47	7.87
UBR4	4	11	0.2538	0.3807	2.16	0.47	7.87
SGK223	4	12	0.2746	0.4092	1.97	0.44	7.04
OBSCN	6	53	0.2976	0.4344	0.57	0.18	1.48
DNAH5	4	13	0.2991	0.4344	1.81	0.41	6.35
PNPLA7	4	13	0.2991	0.4344	1.81	0.41	6.35
XIRP2	4	13	0.2991	0.4344	1.81	0.41	6.35
DNAH10	5	17	0.3447	0.4975	1.75	0.47	5.41
FSIP2	5	18	0.3597	0.5158	1.65	0.45	5.04
ACAN	3	9	0.3986	0.5410	1.94	0.32	8.32
ADH7	3	9	0.3986	0.5410	1.94	0.32	8.32
AVIL	3	9	0.3986	0.5410	1.94	0.32	8.32
CAD	3	9	0.3986	0.5410	1.94	0.32	8.32
CFAP46	3	9	0.3986	0.5410	1.94	0.32	8.32
FAM188B	3	9	0.3986	0.5410	1.94	0.32	8.32
MYO9A	3	9	0.3986	0.5410	1.94	0.32	8.32
SREBF2	3	9	0.3986	0.5410	1.94	0.32	8.32
TOP1MT	3	9	0.3986	0.5410	1.94	0.32	8.32
ACE	3	10	0.4226	0.5506	1.74	0.29	7.24
ADAMTSL4	3	10	0.4226	0.5506	1.74	0.29	7.24
DYNC2H1	3	10	0.4226	0.5506	1.74	0.29	7.24

EFCAB5	3	10	0.4226	0.5506	1.74	0.29	7.24
FASN	3	10	0.4226	0.5506	1.74	0.29	7.24
P2RX7	3	10	0.4226	0.5506	1.74	0.29	7.24
ROS1	3	10	0.4226	0.5506	1.74	0.29	7.24
ATP8B4	3	11	0.4502	0.5640	1.58	0.27	6.40
CDH23	3	11	0.4502	0.5640	1.58	0.27	6.40
CGN	3	11	0.4502	0.5640	1.58	0.27	6.40
FLNC	3	11	0.4502	0.5640	1.58	0.27	6.40
H6PD	3	11	0.4502	0.5640	1.58	0.27	6.40
PKHD1	3	11	0.4502	0.5640	1.58	0.27	6.40
UVSSA	3	11	0.4502	0.5640	1.58	0.27	6.40
OTOG	4	14	0.4895	0.6099	1.68	0.38	5.79
ANK2	4	15	0.5013	0.6112	1.56	0.35	5.30
ANK3	4	15	0.5013	0.6112	1.56	0.35	5.30
COL7A1	4	15	0.5013	0.6112	1.56	0.35	5.30
HELZ2	4	15	0.5013	0.6112	1.56	0.35	5.30
EPPK1	4	16	0.5164	0.6164	1.45	0.33	4.89
FAT2	4	16	0.5164	0.6164	1.45	0.33	4.89
LOXHD1	4	16	0.5164	0.6164	1.45	0.33	4.89
SPTBN5	4	16	0.5164	0.6164	1.45	0.33	4.89
DYSF	4	17	0.5342	0.6344	1.36	0.31	4.54
DNAH14	5	22	0.5733	0.6772	1.32	0.36	3.93
DNAH17	6	27	0.6029	0.7085	1.30	0.40	3.56
LAMA5	6	28	0.6121	0.7157	1.24	0.39	3.41
MYH13	3	12	0.7071	0.8091	1.44	0.25	5.73
NCKAP5	3	12	0.7071	0.8091	1.44	0.25	5.73
UTP20	3	12	0.7071	0.8091	1.44	0.25	5.73
EFCAB6	3	13	0.7162	0.8091	1.32	0.23	5.18
NBAS	3	13	0.7162	0.8091	1.32	0.23	5.18
PKHD1L1	3	13	0.7162	0.8091	1.32	0.23	5.18
CELSR2	3	14	0.7275	0.8091	1.22	0.21	4.72
FLNB	3	14	0.7275	0.8091	1.22	0.21	4.72
FREM2	3	14	0.7275	0.8091	1.22	0.21	4.72
PCNT	3	14	0.7275	0.8091	1.22	0.21	4.72
OTOF	3	15	0.7405	0.8156	1.13	0.20	4.33
SDK1	3	15	0.7405	0.8156	1.13	0.20	4.33
DNAH2	4	18	0.7537	0.8222	1.28	0.30	4.23
PRRC2A	4	18	0.7537	0.8222	1.28	0.30	4.23
DNAH8	4	19	0.7599	0.8250	1.20	0.28	3.95
NEB	5	26	0.7929	0.8517	1.09	0.31	3.19
FBN3	4	28	0.7957	0.8517	0.78	0.19	2.45
USH2A	4	28	0.7957	0.8517	0.78	0.19	2.45
HMCN1	5	34	0.8097	0.8627	0.80	0.23	2.28
AHNAK	5	29	1.0000	1.0000	0.96	0.27	2.79

AKAP9	3	18	1.0000	1.0000	0.93	0.17	3.45
DNAH3	3	19	1.0000	1.0000	0.88	0.16	3.23
DNAH9	3	22	1.0000	1.0000	0.75	0.14	2.70
DST	3	16	1.0000	1.0000	1.06	0.19	3.99
FAT1	3	21	1.0000	1.0000	0.79	0.14	2.86
LAMA3	3	21	1.0000	1.0000	0.79	0.14	2.86
MACF1	5	30	1.0000	1.0000	0.93	0.26	2.67
PDZD2	3	16	1.0000	1.0000	1.06	0.19	3.99
PLEC	5	32	1.0000	1.0000	0.86	0.24	2.46
RNF213	3	18	1.0000	1.0000	0.93	0.17	3.45
SLC27A3	4	21	1.0000	1.0000	1.08	0.25	3.49
SYNE2	3	20	1.0000	1.0000	0.83	0.15	3.03
TNS1	3	18	1.0000	1.0000	0.93	0.17	3.45

Abbreviation: CI, confident interval; NA, not applicable.

[†] The Genes with probable pathgenic variants in at least 3 TOF cases are listed;

[‡] p value was estimated by two-sided Fisher's exact test;

[§] Adjust p value was calculated using Benjamini-Hochberg procedure.

Table S2. The 7 *WDR62* variants identified in TOF patients through WES or Sanger sequencing

cDNA (NM_001083961.1)	Protein (NP_001077430)	Genotype status	Exon	Allele frequency in GnomAD_EAS	Patient ID	SIFT		Polyphen2_HDIV		Polyphen2_HVAR		CADD		WES	Sanger sequencing
						score	pred	score	pred	score	pred	raw	phred		
c.G189T	p.E63D	Heterozygous	2	8.80E-03	A0389	0.042	D	1	D	0.991	D	6.303	29.1	Y	Y
c.A875C	p.N292T	Heterozygous	7	1.50E-03	B530	0.149	T	0.611	P	0.205	B	4.339	24	Y	Y
c.C1441T	p.R481W	Heterozygous	11	3.66E-04	B933	0.002	D	0.268	B	0.051	B	5.49	26.2	Y	Y
c.C1669T	p.R557W	Heterozygous	13	0	B973	0	D	1	D	1	D	7.879	35	Y	Y
c.G1796C	p.C599S	Heterozygous	14	0	B342	0.05	D	0.988	P	0.828	P	5.878	27.6	N	Y
c.T2418G	p.C806W	Heterozygous	20	9.14E-04	B114	0.076	T	1	D	0.999	D	4.379	24.1	N	Y
c.G3287A	p.R1096H	Heterozygous	27	0	B342	0.19	T	1	P	0.999	P	6.317	29.6	N	Y

Abbreviation: GnomAD, Genome Aggregation Database; EAS, East Asian; WES, whole exome sequencing; Y, yes platform performed, N, no sample available.

Table S3. Phenotype analysis of *wdr62*-MO

Heart phenotype	48hpf					72phf				
	Tg(Cmlc2:GFP) n=150		*wdr62*-MO n=150		GFP vs *wdr62*-MO	Tg(Cmlc2:GFP) n=150		*wdr62*-MO n=150		GFP vs *wdr62*-MO
**No loop**	6/150	0.04	9/150	0.06	0.5987	3/150	0.02	48/150	0.32	< 0.0001

**Sinistral loop**	3/150	0.02	21/150	0.14	0.0002	6/150	0.04	12/150	0.08	0.2234
**Cardiac retardation**	0/150	0	108/150	0.72	< 0.0001	0/150	0	69/150	0.46	< 0.0001
**Total**	9/150	0.06	138/150	0.92	< 0.0001	9/150	0.09	129/150	0.86	< 0.0001

The number of zebrafish having heart abnormalities was quantified and percentage was calculated. Significant differences were assessed using a two-sided Fisher's exact test (n = 150, P< 0.050 was considered statistically significant).

Table S4. The gross phenotypes of *Wdr62* knockout mice

Phenotype	WT	HE	*Wdr62*-null	WT vs HE	WT vs *Wdr62*-null
**Developmental retardation**	4/49	14/92	22/45	0.2953	< 0.0001
**Microcephaly**	2/49	7/92	22/45	0.4964	< 0.0001
**Microphthalmia**	0/41	3/76	17/40	0.5508	< 0.0001
**Heart**	4/49	23/92	34/45	0.0231	< 0.0001
**Total**	5/49	30/92	39/45	0.0038	< 0.0001

The number of mice with abnormalities was quantified. Significant differences were assessed using a two-sided Fisher's exact test (P< 0.050 was considered statistically significant).

Table S5. Genotyping of offspring obtained from intercrossing HE mice.

Embryonic day	No. of embryos			
	WT	HE	*Wdr62*-null	Total
**E11**	5<br>(0.23)	12<br>(0.55)	5<br>(0.23)	22<br>(1.00)

**E12**	11 (0.17)	35 (0.56)	17 (0.27)	63 (1.00)
**E13**	13 (0.27)	27 (0.56)	8 (0.17)	48 (1.00)
**E14**	31 (0.36)	45 (0.52)	10 (0.12)	86 (1.00)
**E15**	10 (0.29)	20 (0.59)	4 (0.12)	34 (1.00)
**E16**	6 (0.36)	14 (0.64)	2 (0.10)	22 (1.00)
**P7**	46 (0.30)	92 (0.60)	16 (0.10)	154 (1.00)

Table S6. *Wdr62* knockout mice exhibited a range of defects affecting the heart formation

Phenotype	WT	HE	*Wdr62*-null	WT vs HE	WT vs *Wdr62*-null
**Outflow tract stenosis**	1/49	7/92	21/36	0.2618	< 0.0001
**Short outflow**	1/31	5/60	12/29	0.6598	0.0004
**Great vessel alignment defects**	0/15	4/32	8/13	0.2979	< 0.0001
**Ventricular septal defect**	0/10	3/8	6/6	0.2	0.0003
**Cardiac retardation**	3/45	19/92	21/41	0.0466	< 0.0001
**Total**	4/49	23/92	34/45	0.0231	< 0.0001

The number of mice having heart abnormalities was quantified. Significant differences were assessed using a two-sided Fisher's exact test ($P < 0.050$ was considered statistically significant).

Table S7. Clinical features and targeted sequencing coverage of CHD cases with *WDR62* coding variants

cDNA (NM_001083961.1)	Protein (NP_001077430) n=25	Genotype status	Exon	Allele frequency in our cohort	Allele frequency in GnomAD_EAS	Patient ID n= 50	Gender	Age at diagnosis (month)	Subtype	# of reads for the variant allele	# of reads in total	Variant allele frequency	Sanger seq	WES	Target seq	Risk evaluation[†]
c.2828dupA	p.Y943_S944delinsX	Heterozygous	23	0.0007	0	A1699	Female	5	VSD	3534	7284	0.49	Y	N	Y	Established
c.C3758A	p.S1253X	Heterozygous	30	0.0088	0	B193	Male	4	RVD	848	1883	0.48	Y	N	Y	Established
c.C1669T	p.R557W	Heterozygous	13	0.0021	0	B973	Female	15	TOF	566	1258	0.45	Y	Y	Y	Likely
c.G1796C	p.C599S	Heterozygous	14	0.0025	0	B342	Male	12	TOF	NA	NA	NA	Y	N	Y	Likely
c.G3287A	p.R1096H	Heterozygous	27	0.0025	0	B342	Male	12	TOF	NA	NA	NA	Y	N	Y	Likely
c.A875C	p.N292T	Heterozygous	7	0.0015	0.0015	B530	Male	48	TOF	2468	5124	0.48	Y	Y	Y	Likely
		Heterozygous				A801	Male	72	VSD	2217	4438	0.5	Y	N	Y	
		Heterozygous				B997	Male	10	PA+VSD	1549	3217	0.48	Y	N	Y	
c.C1441T	p.R481W	Heterozygous	11	0.0011	3.66E-04	B933	Male	9	TOF	3757	7798	0.48	Y	Y	Y	Likely
		Heterozygous				A3330	Female	2	VSD	2857	5817	0.49	Y	N	Y	
c.G13C	p.G5R	Heterozygous	1	0.0263	0	B485	Male	36	DORV+PS	918	1858	0.49	Y	N	Y	Likely

c.G781A	p.G261S	Heterozygous	7	0.0263	5.80E-05	B1004	Female	16	DORV+PS	1008	2127	0.47	Y	N	Y	Likely
c.C2515T	p.R839W	Heterozygous	21	0.0014	0	A1851	Male	7	VSD	5301	10844	0.49	Y	N	Y	Likely
		Heterozygous				A1894	Female	36	VSD	4190	8202	0.51	Y	N	Y	
c.G4297A	p.D1433N	Heterozygous	31	0.0091	0	B651	Male	32	PA+VSD	755	1610	0.47	Y	N	Y	Likely
c.G189T	p.E63D	Heterozygous	2	0.0119	0.0088	A0389	NA	NA	TOF	NA	NA	NA	N	Y	N	Uncertain
		Heterozygous				B687	Female	6	TOF	1325	2722	0.49	Y	Y	Y	
		Heterozygous				B804	Male	11	TOF	1190	2405	0.49	Y	N	Y	
		Heterozygous				B847	Female	6	TOF	1436	2905	0.49	Y	N	Y	
		Heterozygous				A1422	Male	4	VSD	1068	2182	0.49	Y	N	Y	
		Heterozygous				A1447	Female	2	VSD	1363	2699	0.51	Y	N	Y	
		Heterozygous				A1577	Male	22	VSD	932	1940	0.48	Y	N	Y	
		Heterozygous				A1944	Female	3	VSD	899	1886	0.48	Y	N	Y	
		Heterozygous				A1983	Female	132	VSD	1131	2372	0.48	Y	N	Y	
		Heterozygous				A636	Female	84	VSD	1317	2749	0.48	Y	N	Y	
		Heterozygous				A749	Male	7	VSD	1146	2408	0.48	Y	N	Y	
		Heterozygous				A1078	Female	5	VSD	1459	3055	0.48	Y	N	Y	
		Heterozygous				A1111	Male	5	VSD	1217	2546	0.48	Y	N	Y	

		Heterozygous				A1836	Male	6	VSD	2284	4796	0.48	Y	N	Y	
		Heterozygous				A1878	Male	7	VSD	985	2079	0.47	Y	N	Y	
		Heterozygous				A3028	Male	23	VSD	1240	2650	0.47	Y	N	Y	
		Heterozygous				A3094	Male	17	VSD	1567	3456	0.45	Y	N	Y	
		Heterozygous				A883	Female	12	VSD	1122	2277	0.49	Y	N	Y	
		Heterozygous				A886	Female	2	VSD	1531	3023	0.51	Y	N	Y	
		Heterozygous				A3333	Male	27	VSD	963	2155	0.45	Y	N	Y	
		Heterozygous				A3370	Female	3	VSD	1267	2640	0.48	Y	N	Y	
		Heterozygous				A3172	Male	3	VSD	1551	3365	0.46	Y	N	Y	
		Heterozygous				A2667	Female	18	RVD	910	1968	0.46	Y	N	Y	
		Heterozygous				A2857	Male	5	RVD	988	1976	0.5	Y	N	Y	
		Heterozygous				B80	Male	65	RVD	1494	2969	0.5	Y	N	Y	
		Heterozygous				B519	Male	8	RVD	1140	2271	0.5	Y	N	Y	
c.C80T	p.A27V	Heterozygous	1	0.0007	0	A1828	Female	6	VSD	457	2231	0.2	Y	N	Y	Uncertain
c.G187A	p.E63K	Heterozygous	2	0.0007	0	A3093	Male	8	VSD	990	2252	0.44	Y	N	Y	Uncertain
c.G253A	p.V85M	Heterozygous	2	0.0007	0	A1635	Male	6	VSD	936	1892	0.49	Y	N	Y	Uncertain
c.G596A	p.C199Y	Heterozygous	6	0.0007	0	A761	Female	36	VSD	1155	2364	0.49	Y	N	Y	Uncertain
c.C704T	p.T235M	Heterozygous	7	0.0007	0	A1654	Female	13	VSD	881	1773	0.5	Y	N	Y	Uncertain

c.A758T	p.N253I	Heterozygous	7	0.0007	0	A706	Male	3	VSD	2935	5969	0.49	Y	N	Y	Uncertain
c.C1009A	p.L337I	Heterozygous	8	0.0007	0	A1632	Male	4	VSD	636	1440	0.47	Y	N	Y	Uncertain
c.G1277C	p.G426A	Heterozygous	10	0.0007	0	A679	Female	7	VSD	1359	2681	0.51	Y	N	Y	Uncertain
c.C1948T	p.R650C	Heterozygous	15	0.0007	0	A569	Female	24	VSD	1529	2949	0.52	Y	N	Y	Uncertain
c.T2418G	p.C806W	Heterozygous	20	0.0013	9.14E-04	B114	Female	5	TOF	NA	NA	NA	Y	N	Y	Uncertain
		Heterozygous				A732	Female	5	VSD	1630	1675	0.97	Y	N	Y	
c.3167_3169del	p.1056_1057del	Heterozygous	26	0.0007	0	A3067	Female	3	VSD	1036	2060	0.5	Y	N	Y	Uncertain
c.C3302T	p.T1101M	Heterozygous	27	0.0007	9.14E-05	A3008	Male	10	VSD	689	1383	0.5	Y	N	Y	Uncertain
c.C4354T	p.R1452W	Heterozygous	32	0.0014	5.81E-05	A1174	Male	12	VSD	1281	2586	0.5	Y	N	Y	Uncertain
		Heterozygous				A1926	Female	24	VSD	901	1864	0.48	Y	N	Y	

A total of 55 of 1320 cases were found carrying *WDR62* variants in coding region (4.17 %).

Variants with the following features are listed: a) non-synonymous coding variants; b) MAF < 1% in the GnomAD_EAS exomes or exome and genome collections (see Table S8); c) CADD Phred score> 20 (top 1% deleterious) without tolerated/ benign status by SIFT/Polyphen2 (see Table S9) or indel variants. Abbreviation: GnomAD, Genome Aggregation Database; EAS, East Asian; WES, whole exome sequencing; Target seq, Target sequencing; Y, yes platform performed, N, no sample available. NA, no data available.

† Variants identified in this study were classified and described as “established risk allele”, “likely risk allele” or “uncertain risk allele” according to ACMG laboratory guideline.

Table S8. MAF for all *WDR62* variants from the 1000G_2015aug, ESP6500SI-V2, ExAC and GnomAD

cDNA	Protein	1000G_2015aug		ESP6500SI-V2	ExAC		GnomAD_exome		GnomAD_genome	
c.G13C	p.G5R	0	0	0	5.83E-05	0	1.76E-05	0	0	0
c.C80T	p.A27V	0	0	0	0	0	0	0	0	0
c.G187A	p.E63K	0	0	0	0	0	4.06E-06	0	0	0
c.G189T	p.E63D	0.0008	0.004	0	0.0006	0.0085	0.0007	0.0089	0.0004	0.0077
c.G253A	p.V85M	0.0002	0	0.0025	0.0012	0	0.0012	0	0.0014	0
c.G596A	p.C199Y	0	0	0	8.27E-06	0	4.06E-06	0	0	0
c.C704T	p.T235M	0	0	0	0	0	8.14E-06	0	0	0
c.A758T	p.N253I	0	0	0	0	0	0	0	0	0
c.G781A	p.G261S	0	0	0	0	0	3.98E-06	5.44E-05	0	0
c.A875C	p.N292T	0	0	0	0.0001	0.0018	0.0001	0.0015	9.56E-05	0.0019
c.C1009A	p.L337I	0	0	0	0	0	0	0	0	0
c.G1277C	p.G426A	0.0002	0.001	0	0	0	3.98E-06	5.44E-05	0	0
c.C1441T	p.R481W	0	0	0	2.77E-05	0.0002	4.40E-05	0.0004	9.55E-05	0
c.C1669T	p.R557W	0	0	0	0	0	4.37E-06	0	0	0

**c.G1796C**	**p.C599S**	**0**	**0**	**0**	**0**	**0**	**0**	**0**	**0**	**0**
**c.C1948T**	**p.R650C**	**0**	**0**	**0**	**1.65E-05**	**0**	**8.13E-06**	**0**	**0**	**0**
c.T2418G	p.C806W	0.001	0.003	0	0.0001	0.0009	9.54E-05	0.0009	0	0
**c.C2515T**	**p.R839W**	**0**	**0**	**7.70E-05**	**0**	**0**	**0**	**0**	**9.68E-05**	**0**
**c.2828dupA**	**p.Y943_S944delinsX**	**0**	**0**	**0**	**0**	**0**	**0**	**0**	**0**	**0**
**c.3167_3169del**	**p.1056_1057del**	**0**	**0**	**0**	**0**	**0**	**4.06E-06**	**0**	**0**	**0**
**c.G3287A**	**p.R1096H**	**0**	**0**	**0.0002**	**0.000049**	**0**	**0.000048**	**0**	**0**	**0**
**c.C3302T**	**p.T1101M**	**0**	**0**	**7.70E-05**	**3.30E-05**	**0.0001**	**5.17E-05**	**0.0001**	**9.56E-05**	**0**
**c.C3758A**	**p.S1253X**	**0**	**0**	**0**	**0**	**0**	**0**	**0**	**0**	**0**
**c.G4297A**	**p.D1433N**	**0**	**0**	**0**	**0**	**0**	**1.22E-05**	**0**	**0**	**0**
**c.C4354T**	**p.R1452W**	**0**	**0**	**7.70E-05**	**1.69E-05**	**0.0001**	**1.20E-05**	**0.0001**	**3.19E-05**	**0.0006**
**-112_-107delCCCCGC**		**0**	**0**	**0**	**0**	**0**	**0**	**0**	**0**	**0**
**-106C>G**		**0**	**0**	**0**	**0**	**0**	**0**	**0**	**0**	**0**
-65G>C		0.0004	0.002	0	0	0	0	0	3.19E-05	0.0006
**-23G>A**		**0.0002**	**0.001**	**0**	**0**	**0**	**0**	**0**	**3.23E-05**	**0.0006**
**c.-82G>A**		**0**	**0**	**0**	**0**	**0**	**0**	**0**	**0**	**0**
**c.-6G>C**		**0**	**0**	**0**	**0**	**0**	**0**	**0**	**0**	**0**
c.333-6C>A		0	0	0	3.30E-05	0.0005	7.95E-05	0.0011	9.56E-05	0.0019

**c.561+10G>A**	0	0	7.70E-05	2.20E-05	0	1.54E-05	0.0001	0	0
**c.1642+9G>A**	0.0002	0.001	7.70E-05	1.66E-05	0.0002	2.00E-05	0.0001	0	0
**c.1958+6G>A**	0	0	0	0	0	0	0	0	0
**c.1958+8G>T**	0	0	0	0	0	0	0	0	0
**c.2868-18_2868-7del**	0	0	0	0	0	0	0	0	0
**c.2868-6C>G**	0	0	0	0	0	0	0	0	0
c.3083-6T>C	0.0004	0.002	0	3.31E-05	0.0005	3.58E-05	0.0005	9.56E-05	0.0019
**c.3220+4G>C**	0.0002	0.001	0	4.99E-05	0.0007	6.38E-05	0.0009	0	0
**c.3462+8T>C**	0	0	0	8.27E-06	0	1.19E-05	0	0	0
**c.4311+8A>C**	0	0	0	0	0	0	0	0	0
**c.*36G>C**	0	0	0	0	0	0	0	0	0
***7G>A**	0	0	0	0	0	0	0	0	0
***14G>A**	0	0	0	0	0	0	0	0	0
***16T>C**	0	0	0	0	0	0	0	0	0
***46G>C**	0	0	0	0	0	0	0	0	0

Variants with bold font were absent from East Asian population (n= 19) or at extremely low frequency (≤ 0.1%, n= 21) in GnomAD, 1000G and ExAC (PM2).

Boxes around the MAF correspond to the EAS population group.

Red number indicated that the variants were completely absent from its corresponding population.

Table S9. The predicting deleterious effects on encoded amino acids for all missense variants

cDNA	Protein	SIFT		Polyphen2_HDIV		Polyphen2_HVAR		MutationTaster		MutationAssessor		CADD	
		score	pred	score	pred	score	pred	score	pred	score	pred	raw	phred
**c.G13C**	**p.G5R**	0.002	D	0.996	D	0.925	D	0.842	D	2.015	M	5.237	25.6
c.C80T	p.A27V	0	D	0.995	D	0.794	P	0.839	N	2.14	M	3.906	23.5
c.G187A	p.E63K	0.054	T	0.996	D	0.888	P	1	D	2.19	M	7.536	34
**c.G189T**	**p.E63D**	0.042	D	1	D	0.991	D	0.999	D	2.19	M	6.303	29.1
**c.G253A**	**p.V85M**	0.002	D	0.993	D	0.865	P	0.964	D	2.82	M	5.575	26.4
c.G596A	p.C199Y	0.021	D	0.999	D	0.985	D	1	D	1.1	L	6.215	28.7
c.C704T	p.T235M	0.009	D	1	D	0.998	D	0.61	N	1.67	L	5.357	25.9
**c.A758T**	**p.N253I**	0	D	0.999	D	0.973	D	0.97	D	2.83	M	5.555	26.4
c.G781A	p.G261S	0.056	T	1	D	0.982	D	1	D	2.565	M	7.082	33
c.A875C	p.N292T	0.149	T	0.611	P	0.205	B	0.994	D	1.04	L	4.339	24
**c.C1009A**	**p.L337I**	0.005	D	1	D	0.998	D	1	D	2.585	M	5.631	26.6
c.G1277C	p.G426A	0.061	T	1	D	1	D	1	D	2.725	M	5.244	25.6
c.C1441T	p.R481W	0.002	D	0.268	B	0.051	B	0.956	D	1.43	L	5.49	26.2
**c.C1669T**	**p.R557W**	0	D	1	D	1	D	1	D	2.69	M	7.879	35
**c.G1796C**	**p.C599S**	0.05	D	0.988	P	0.828	P	1	D	2.725	M	5.878	27.6

**c.C1948T**	**p.R650C**	0	D	1	D	0.901	P	1	D	2.07	M	7.737	35
c.T2418G	p.C806W	0.076	T	1	D	0.999	D	0.999	D	2.4	M	4.379	24.1
**c.C2515T**	**p.R839W**	0	D	1	D	0.996	D	0.996	D	2.48	M	7.846	35
c.G3287A	p.R1096H	0.19	T	1	P	0.999	P	0.793	D	1.545	L	6.317	29.6
c.C3302T	p.T1101M	0.001	D	0.999	D	0.891	P	0.963	N	0.55	N	6.409	29.6
c.G4297A	p.D1433N	0.016	D	0.998	D	0.857	P	0.88	N	2.015	M	3.202	22.7
c.C4354T	p.R1452W	0.012	D	0.996	D	0.702	P	1	N	1.59	L	6.052	28

Variants with the CADD Phred score> 20 and Damaging/ Possibly Damaging status by SIFT/Polyphen2 are listed, and variants with bold font were predicted to be deleterious.

Categorical predictions for each Algorithm. SIFT: "T"= Tolerated, "D"= Damaging. PolyPhen2 HDIV & HVAR: "B"= Benign, "P"= Possibly Damaging, "D"= Probably Damaging. MutationTaster: "N"= Polymorphisms, "D"= Disease-causing. MutationAssessor: "M" & "H"= Functional, "L" & "N"= Non-functional.

Table S10. Algorithms predicting nucleotide conservation

cDNA	Protein	GERP++_RS	PhyloP	PhastCons-100 way
**c.G13C**	**p.G5R**	5.21	3.03	0.99
**c.C80T**	**p.A27V**	5.18	3.03	1
**c.G187A**	**p.E63K**	5.58	5.61	1
**c.G189T**	**p.E63D**	5.58	2.14	1
c.G253A	p.V85M	4.49	0.69	0.55

**c.G596A**	**p.C199Y**	5.51	5.61	1
**c.C704T**	**p.T235M**	4.73	2.38	0.98
**c.A758T**	**p.N253I**	5.76	1.42	0.91
**c.G781A**	**p.G261S**	5.76	5.61	1
**c.A875C**	**p.N292T**	5.71	4.73	1
**c.C1009A**	**p.L337I**	3.84	3.11	1
**c.G1277C**	**p.G426A**	5.23	2.55	1
c.C1441T	p.R481W	4.67	0.77	0.96
**c.C1669T**	**p.R557W**	5.31	1.82	1
**c.G1796C**	**p.C599S**	5.49	5.45	1
**c.C1948T**	**p.R650C**	3.44	2.22	1
c.T2418G	p.C806W	0.513	0.04	0.99
**c.C2515T**	**p.R839W**	5.03	2.06	1
c.2828dupA	p.Y943_S944delinsX		-0.92	0.02
**c.3167_3169del**	**p.1056_1057del**		0.04~2.79	0.99~1
**c.G3287A**	**p.R1096H**	5.29	3.03	0.96
c.C3302T	p.T1101M	4.24	1.74	0.81
c.C3758A	p.S1253X	-0.066	0.02	0.14

c.G4297A	p.D1433N	2.59	0.85	0.96
c.C4354T	p.R1452W	5.21	3.03	0.06
-112_-107delCCCCGC			-4.07~1.5	0~0.03
-106C>G			0.04	0
-65G>C			-0.84	0
-23G>A			-0.12	0.01
**c.-82G>A**			3.03	0.99
c.-6G>C			0.45	0
**c.333-6C>A**			1.5	0.99
c.561+10G>A			-1.81	0
c.1642+9G>A			-2.38	0
c.1958+6G>A			0.37	0.45
c.1958+8G>T			0.45	0.05
c.2868-18_2868-7del			-<br>2.46~1.01	0~0.02
c.2868-6C>G			-0.04	0
c.3083-6T>C			-0.92	0
c.3220+4G>C			0.69	0.87

c.3462+8T>C	-0.52	0.01
c.4311+8A>C	-1.17	0
c.*36G>C	0.04	0
*7G>A	0.12	0
*14G>A	-1.97	0
*16T>C	0.61	0
*46G>C	2.55	0.04

PHASTCons and GERP++ Elements provide estimates of evolutionary conservation as to whether the variant appears as part of a larger conserved element or region.

GERP++ RS, PhyloP scores predict conservation at a nucleotide-by-nucleotide basis.

Variants meets both PhyloP>1 and PhastCons-100 way>0.9 are listed in bold and were predicted to be nucleotide conserved.

Table S11. Clinical features and targeted sequencing coverage of CHD cases with *WDR62* coding variants

Mutation Information		Genotype status	Allele frequencies in our cohort	Allele frequencies from GnomAD_EAS	Patient ID n= 25	Gender	Age at diagnosis (month)	Subtype	# of reads for the variant allele	# of reads in total	Variant allele frequency	Sanger seq	Risk allele†
***WDR62* variants n=22**	**Position (hg19/chr7)**												
c.-6G>C	36545868	Heterozygous	0.001	0	A1453	Male	53	TOF	1766	3663	0.48	N	Likely
		Heterozygous			A3056	Female	2	VSD	1896	3939	0.48	N	Likely
c.2868-18_2868-7delCCCCCACTGGCA	36592098-36592109	Heterozygous	0.0021	0	A3093	Male	8	VSD	954	2210	0.43	Y	Likely
		Heterozygous			A568	Female	12	VSD	994	2264	0.44	Y	Likely
		Heterozygous			K357	Male	10	VSD	1014	2269	0.45	Y	Likely

c.2868-6C>G	36592110	Heterozygous	0.0021	0	A3093	Male	8	VSD	992	2265	0.44	Y	Likely
		Heterozygous			A568	Female	12	VSD	992	2265	0.44	Y	Likely
		Heterozygous			K357	Male	10	VSD	1012	2270	0.45	Y	Likely
*14G>A	36596026	Heterozygous	0.0089	0	B938	Female	10	PA+VSD	826	1730	0.48	Y	Likely
		Heterozygous			B414	Male	12	RVD	1274	2654	0.48	N	
-112_-107delCCCCGC	36545671-36545676	Heterozygous	0.0021	0	B1128	Female	8	TOF	1093	2375	0.46	Y	Uncertain
-65G>C	36545718	Heterozygous	0.0021	5.01E-05	A1471	Male	5	VSD	1015	2377	0.43	N	Uncertain
c.-82G>A	36545792	Heterozygous	0.0021	0	B476	Male	48	TOF	2532	5104	0.5	N	Uncertain
c.561+10G>A	36557339	Heterozygous	0.0014	1.43E-04	A1416	Female	2	VSD	1925	3935	0.49	Y	Uncertain
		Heterozygous			A1917	Male	36	VSD	2520	4783	0.53	Y	Uncertain
-106C>G	36545677	Heterozygous	0.0007	0	A1471	Male	5	VSD	895	1768	0.51	N	Uncertain
-23G>A	36545760	Heterozygous	0.0007	5.01E-05	A3272	Male	30	VSD	702	1468	0.48	N	Uncertain
c.333-6C>A	36556854	Heterozygous	0.0021	9.52E-04	B738	Female	16	TOF	1269	2679	0.47	Y	Uncertain
c.1642+9G>A	36575655	Heterozygous	0.0007	1.09E-04	A777	Male	10	VSD	853	1696	0.5	Y	Uncertain
c.1958+6G>A	36580214	Heterozygous	0.0007	0	A1862	Male	13	VSD	1324	2624	0.5	Y	Uncertain
c.1958+8G>T	36580216	Heterozygous	0.0007	0	A1694	Male	72	VSD	1021	2055	0.5	Y	Uncertain
c.3083-6T>C	36592910	Heterozygous	0.0007	6.02E-04	A877	Male	15	VSD	1638	3272	0.5	Y	Uncertain
c.3220+4G>C	36593057	Heterozygous	0.001	8.75E-04	A3009	Female	2	VSD	1013	1972	0.51	Y	Uncertain
		Heterozygous			B601	Male	1	TOF	1608	3337	0.48	Y	Uncertain
c.3462+8T>C	36593984	Heterozygous	0.0007	0	A2118	Male	4	VSD	827	1761	0.47	Y	Uncertain
c.4311+8A>C	36595585	Heterozygous	0.0007	0	A778	Male	6	VSD	1152	2400	0.48	Y	Uncertain
c.*36G>C	36595966	Heterozygous	0.0007	0	A1167	Male	2	VSD	1470	3044	0.48	Y	Uncertain
*7G>A	36596019	Heterozygous	0.0007	0	A3278	Female	72	VSD	1559	3155	0.49	Y	Uncertain
*16T>C	36596028	Heterozygous	0.0007	0	A3132	Male	8	VSD	1360	2651	0.51	Y	Uncertain
*46G>C	36596058	Heterozygous	0.0007	0	A3103	Male	0	VSD	1294	2503	0.52	Y	Uncertain
A total of 26 of 1083 cases were found carrying *WDR62* non-coding variants (2.03%)													

The listed variants are with MAF< 1% in the GnomAD_EAS exomes or exome and genome collections (see Table S8).

[†] Variants identified in this study were classified and described as “established risk allele”, “likely risk allele” or “uncertain risk allele” according to ACMG laboratory guideline.

Table S12. Prediction of variant effects on splicing consensus

cDNA	Predicted Signal (Number of algorithms predicting result)		
	**Donor (4)**	**Acceptor (4)**	**Enhancers (2)**
c.333-6C>A		Site Broken (4)	ESE site Broken (1)
c.561+10G>A			ESE site Broken (2)
c.1642+9G>A	Site Broken (1)		ESE site Broken (1)
c.1958+6G>A	Site Broken (1) New Site (2)		ESE site Broken (2)
c.1958+8G>T	New Site (1)		
c.2868-18_2868-7del	Site Broken (4) New Site (4)	New Site (1)	ESE site Broken (2)
c.2868-6C>G		Site Broken (3) New Site (2)	ESE site Broken (2)
c.3083-6T>C		Site Broken (3)	ESE site Broken (1)
c.3220+4G>C			ESE site Broken (1)
c.3462+8T>C		New Site (1)	ESE site Broken (1)
c.4311+8A>C	New Site (1)		

Abbreviation: ESE, exonic splicing enhancer sites.

Variant sites located in introns were performing splicing analysis. Algorithms predicting splicing signals includes SpliceSiteFinder, MaxEntScan, GeneSplicer and NNSPLICE; Algorithms predicting ESE binding site includes ESEFinder and RESCUE-ESE.

Table S13. Prediction of variant effects on regulatory elements

cDNA	Start	End	ChIP data (heart tissue)	Chromatin state (heart tissue)	Accessibility (heart tissue)	Motif (PWM)	Probability	Ranking
**-112_-107delCCCCGC**	36545671	36545676	CTCF (H)	Active TSS (H, RV, LV, RA)	6 (H), 1 (RV), 3 (LV)	KLF4 (TSTFF908150), SP1 (TSTFF587073, TSTFF397036, TSTFF817518, TSTFF618931, TSTFF636026, TSTFF283590, TSTFF245752), SP4 (TSTFF954734)	0.13~1	2a
-106C>G	36545677	36545677	CTCF (H)	Active TSS (H, RV, LV, RA)	8 (H), 1 (RV), 3 (LV)		0.7	4
**-65G>C**	36545718	36545718	CTCF, TAF1	Active TSS (H, RV, LV, RA)	8 (H), 1 (RV), 3 (LV)	HIC1 (TSTFF331059)	0.84	2b
-23G>A	36545760	36545760	CTCF, POLR2A	Active TSS (H, RV, LV, RA)	8 (H), 1 (RV), 3(LV)		0.61	4
c.-82G>A	36545792	36545792	MXI1, PHF8	Active TSS (H, RV, LV, RA)	3(H)		0.61	4
c.-6G>C	36545868	36545868	MXI1, PHF8	Active TSS (H, RV, LV, RA)	1 (IPS)		0.61	4

c.*36G>C	36595966	36595966	PRDM2, AGO2	Strong transcription (LV, RA), Enhancers (RV), Quiescent/Low (H)	1 (IPS)		0.61	4
*7G>A	36596019	36596019	AGO2, REST	Strong transcription (LV, RA), Enhancers (RV), Quiescent/Low (H)	1 (IPS)		0.61	4
*14G>A	36596026	36596026	AGO2, REST	Strong transcription (LV, RA), Enhancers (RV), Quiescent/Low (H)	1 (IPS)		0.61	4
*16T>C	36596028	36596028	AGO2, REST	Strong transcription (LV, RA), Enhancers (RV), Quiescent/Low (H)	1 (IPS)		0.61	4

***46G>C**	36596058	36596058	AGO2, REST	Strong transcription (LV, RA), Enhancers (RV), Quiescent/Low (H)	1 (IPS)	EWSR1 (TSTFF589502), NKX2-3 (TSTFF811418, TSTFF215857), NKX2-8 (TSTFF470165, TSTFF700026)	0.62	2b

Abbreviation: H, heart; RV, right ventricle; LV, left ventricle; RA, right atrium; IPS, induced Pluripotent stem cells. PWM, position-weight matrix for TF binding.

The regulatory effect of variants located in WDR62 non-coding regulatory region was predicted by RegulomeDB v2.0.

The listed regulatory DNA elements include regions of DNase hypersensitivity (see the DNase Footprinting results in "Accessibility"), binding sites of transcription factors (see "ChIP data" and "Motif"), and promoter regions that have been biochemically characterized to regulation transcription (see "Chromatin state"). Results from heart tissue are marked in brackets. The unmarked results were from other tissue types including pluripotent stem cells, brain, kidney, liver or blood.

Variants with Ranking "1" or "2" and Probability >0.5 are listed in bold and were predicted to be located on regulatory DNA elements.

Table S14. The predicted effects of variant in the 3'UTR (c.*36G>C) on predicted microRNA binding sites

**Removed (wild-type)**					
**miRNA name**	**Position in 3' UTR**	**Alignment Length**	**Alignment Score**	**Identity**	**Similarity**
hsa-miR-4330	c.*21_*39	6	120	100.00%	100.00%
**Modified (wild-type ⇒ mutant)**					
**miRNA name**	**Position in 3' UTR**	**Alignment Length**	**Alignment Score**	**Identity**	**Similarity**
hsa-miR-330-3p (1)	c.*14_*37 ⇒ c.*14_*36	21 ⇒ 18	125.00 ⇒ 121.00	76.19% ⇒ 77.78%	76.19% ⇒ 77.78%
hsa-miR-875-5p	c.*31_*50 ⇒ c.*29_*50	19	133.00 ⇒ 132.00	68.42% ⇒ 63.16%	84.21% ⇒ 78.95%

Added (mutant)					
**miRNA name**	**Position in 3' UTR**	**Alignment Length**	**Alignment Score**	**Identity**	**Similarity**
hsa-miR-1200	c.*20_*41	7	140	100.00%	100.00%
hsa-miR-125a-5p	c.*18_*41	7	125	100.00%	100.00%
hsa-miR-125b	c.*20_*41	7	125	100.00%	100.00%
hsa-miR-378*	c.*20_*41	6	120	100.00%	100.00%
hsa-miR-4324	c.*21_*40	7	140	100.00%	100.00%
hsa-miR-4329	c.*21_*39	6	120	100.00%	100.00%
hsa-miR-580	c.*17_*38	6	120	100.00%	100.00%

The analysis of microRNA binding sites was performed using Alamut software and the report is based on miranda prediction and microRNA. org targets. For "Added miRNA", only the miRNA of which the identity and Similarity were 100.00% are listed.

Table S15. Case-Control Association Studies: observed allele counts for all variants screened in our index cases and the GnomAD reference database

cDNA	Protein	Number_Subtype	Index Cases Allele Counts		gnomAD_EAS Allele Counts		OR	p value	P.adj.BH
**c.G13C**	**p.G5R**	**1 DORV+PS**	**1**	**38**	**0**	**17176**	**Inf**	**0.0022**	**0.022325**
c.C80T	p.A27V	1_VSD	1	1436	0	19954	Inf	0.0707	0.09722286
c.G187A	p.E63K	1_VSD	1	1436	0	18388	Inf	0.0724	0.09722286

c.G189T	p.E63D	4_TOF, 18_VSD, 4_RVD	26	2492	176	19950	1.185	0.4301	0.459425
c.G253A	p.V85M	1_VSD	1	1436	0	19954	Inf	0.0671	0.09556667
c.G596A	p.C199Y	1_VSD	1	1436	0	18392	Inf	0.0724	0.09556667
c.C704T	p.T235M	1_VSD	1	1436	0	18392	Inf	0.0724	0.09556667
c.A758T	p.N253I	1_VSD	1	1436	0	18392	Inf	0.0724	0.09556667
**c.G781A**	**p.G261S**	**1_DORV+PS**	**1**	**38**	**1**	**18394**	**497.1**	**0.0041**	**0.0334875**
c.A875C	p.N292T	1_TOF, 1_VSD, 1_PA+VSD	3	2488	30	19890	0.7992	>0.9999	1
c.C1009A	p.L337I	1_VSD	1	1436	0	18394	Inf	0.0724	0.09556667
c.G1277C	p.G426A	1_VSD	1	1436	1	18394	12.82	0.1396	0.17266316
c.C1441T	p.R481W	1_TOF,1_VSD	2	2378	7	19930	2.4	0.248	0.28429268
c.C1669T	p.R557W	1_TOF	1	942	0	19954	Inf	0.0451	0.09556667
c.G1796C	p.C599S	1_TOF	1	942	0	18394	Inf	0.0487	0.09556667
c.C1948T	p.R650C	1_VSD	1	1436	0	18392	Inf	0.0724	0.09556667
c.T2418G	p.C806W	1_TOF, 1_VSD	2	2378	17	19954	0.987	>0.9999	1
**c.C2515T**	**p.R839W**	**1_VSD**	**2**	**1436**	**0**	**19954**	**Inf**	**0.005**	**0.0334875**
c.2828dupA	p.Y943_S944delinsX	1_VSD	1	1436	0	18372	Inf	0.0725	0.09556667
c.3167_3169del	p.1056_1057del	1_VSD	1	1436	0	18394	Inf	0.0724	0.09556667
c.G3287A	p.R1096H	1_TOF	1	942	0	18394	Inf	0.0487	0.09556667

c.C3302T	p.T1101M	1_VSD	1	1436	2	19952	6.951	0.1882	0.22680513
**c.C3758A**	**p.S1253X**	1_RVD	**1**	**114**	**0**	**19954**	**Inf**	**0.0057**	**0.0334875**
**c.G4297A**	**p.D1433N**	**1_VSD+PA**	**1**	**110**	**0**	**18388**	**Inf**	**0.0059**	**0.0334875**
c.C4354T	p.R1452W	2_VSD	2	1436	3	19920	9.259	0.0394	0.09556667
-112_-107delCCCCGC		1_TOF	1	468	0	19954	Inf	0.0229	0.08969167
-106C>G		1_VSD	1	1436	0	19954	Inf	0.0769	0.09768378
-65G>C		1_TOF	1	468	1	19954	Inf	0.0229	0.08969167
-23G>A		1_VSD	1	1436	1	19954	Inf	0.0769	0.09768378
c.-82G>A		1_TOF	1	468	0	19954	Inf	0.0229	0.08969167
**c.-6G>C**		**1_VSD, 1_TOF**	**2**	**1904**	**0**	**16096**	**Inf**	**0.0112**	**0.03968889**
c.333-6C>A		1_TOF	1	468	19	19950	2.246	0.3712	0.41539048
c.561+10G>A		2_VSD	2	1436	2	14034	9.785	0.0455	0.09556667
c.1642+9G>A		1_VSD	1	1436	2	18386	6.406	0.202	0.23735
c.1958+6G>A		1_VSD	1	1436	0	18388	Inf	0.0724	0.09556667
c.1958+8G>T		1_VSD	1	1436	0	18388	Inf	0.0724	0.09556667
**c.2868-18_2868-7del**		**3_VSD**	**3**	**1436**	**0**	**19214**	**Inf**	**0.0003**	**0.0047**
**c.2868-6C>G**		**3_VSD**	**3**	**1436**	**0**	**17924**	**Inf**	**0.0003**	**0.0047**
c.3083-6T>C		1_VSD	1	1436	12	19946	1.158	0.5951	0.62154889

c.3220+4G>C	1_TOF, 1_VSD	2	1904	16	18296	1.602	0.3857	0.42157907
c.3462+8T>C	1_VSD	1	1436	0	18390	Inf	0.0724	0.09556667
c.4311+8A>C	1_VSD	1	1436	0	18386	Inf	0.0724	0.09556667
c.*36G>C	1_VSD	1	1436	0	15642	Inf	0.0841	0.09556667
*7G>A	1_VSD	1	1436	0	19954	Inf	0.0769	0.09556667
***14G>A**	**1_PA+VSD, 1_RVD**	**2**	**224**	**0**	**19954**	**Inf**	**0.0001**	**0.0047**
*16T>C	1_VSD	1	1436	0	19954	Inf	0.0769	0.09556667
*46G>C	1_VSD	1	1436	0	19954	Inf	0.0769	0.09556667

Abbreviation: Inf, Infinite.

Observed allele counts for all variants screened in our index cases and the exomes or exome and genome collections of allele counts in the GnomAD_EAS reference database.

An OR of 1 indicates no difference between the frequency of cases and controls. The nominal p-value is the probability that the observed OR is greater than expected by random chance (> 1), using a two-sided Fisher’s exact test (P< 0.050 was considered statistically significant).

Variants with OR> 5 and P< 0.050 (n= 16) are listed in bold (PS4).

Table S16. Criteria assessed during this study on each of the 47 *WDR62* variants

cDNA	Protein	MAF	Case-control	Deleterious effects on amino acids	Nucleotide Conservation	Splicing Consensus	Predicted regulatory elements	3'UTR MicroRNA Binding	Experimental Functional studies

c.G13C	p.G5R	√	√	√	√	
c.C80T	p.A27V	√	□	□	√	
c.G187A	p.E63K	√	□	□	√	
c.G189T	p.E63D	□	□	√	√	√
c.G253A	p.V85M	√	□	√	□	
c.G596A	p.C199Y	√	□	□	√	
c.C704T	p.T235M	√	□	□	√	
c.A758T	p.N253I	√	□	√	√	
c.G781A	p.G261S	√	√	□	√	
c.A875C	p.N292T	□	□	□	√	√
c.C1009A	p.L337I	√	□	√	√	
c.G1277C	p.G426A	√	□	□	√	
c.C1441T	p.R481W	√	□	□	□	√
c.C1669T	p.R557W	√	□	√	√	√
c.G1796C	p.C599S	√	□	√	√	√
c.C1948T	p.R650C	√	□	√	√	
c.T2418G	p.C806W	□	□	□	□	√
c.C2515T	p.R839W	√	√	√	√	

c.2828dupA	p.Y943_S944delinsX	√	□		□			
c.3167_3169del	p.1056_1057del	√	□		√			
c.G3287A	p.R1096H	√	□	□	√			√
c.C3302T	p.T1101M	√	□	□	□			
c.C3758A	p.S1253X	√	√		□			
c.G4297A	p.D1433N	√	√	□	□			
c.C4354T	p.R1452W	√	□	□	□			
-112_-107delCCCCGC		√	□		□		√	
-106C>G		√	□		□		□	
-65G>C		□	□		□		√	
-23G>A		√	□		□		□	
c.-82G>A		√	□		√		□	
c.-6G>C		√	√		□		□	
c.333-6C>A		□	□		√	√		
c.561+10G>A		√	□		□	√		
c.1642+9G>A		√	□		□	√		
c.1958+6G>A		√	□		□	√		

c.1958+8G>T	√	□	□	√		
c.2868-18_2868-7del	√	√	□	√		
c.2868-6C>G	√	√	□	√		
c.3083-6T>C	□	□	□	√		
c.3220+4G>C	√	□	□	√		
c.3462+8T>C	√	□	□	√		
c.4311+8A>C	√	□	□	√		
c.*36G>C	√	□	□		□	√
*7G>A	√	□	□		□	
*14G>A	√	√	□		□	
*16T>C	√	□	□		□	
*46G>C	√	□	□		√	

305 **Table S17. Summaries of the ACMG-AMP evidentiary criteria for all *WDR62* variants**

Risk allele‡	cDNA	Protein	Pathogenic Criteria																Benign Criteria											
				Strong				Moderate						Supporting						Strong				Supporting						
			PVS1	PS1	PS2	PS3	PS4	PM1†	PM2	PM3	PM4	PM5†	PM6	PP1	PP2	PP3	PP4	PP5	BA1	BS1	BS2	BS3	BS4	BP1	BP2	BP3	BP4	BP5	BP6	BP7

Likely	c.G13C	p.G5R		√		√	√		
Uncertain	c.C80T	p.A27V				√	√		
Uncertain	c.G187A	p.E63K				√	√		
Uncertain	c.G189T	p.E63D	√				√		
Uncertain	c.G253A	p.V85M			√	√	√		
Uncertain	c.G596A	p.C199Y			√	√	√		
Uncertain	c.C704T	p.T235M			√	√	√		
Uncertain	c.A758T	p.N253I				√	√		
Likely	c.A875C	p.N292T	√		√		√		√
Likely	c.G781A	p.G261S		√		√	√		
Uncertain	c.C1009A	p.L337I			√	√	√		
Uncertain	c.G1277C	p.G426A			√	√	√		
Likely	c.C1441T	p.R481W	√		√	√			
Likely	c.C1669T	p.R557W	√		√	√	√		
Likely	c.G1796C	p.C599S	√		√	√	√		
Uncertain	c.C1948T	p.R650C			√	√	√		
Uncertain	c.T2418G	p.C806W	√						
Likely	c.C2515T	p.R839W		√		√	√	√	

Estabished	c.2828dupA	p.Y943_S944delinsX	√			√		√			
Uncertain	c.3167_3169del	p.1056_1057del				√	√	√			
Likely	c.G3287A	p.R1096H		√		√		√			
Uncertain	c.C3302T	p.T1101M				√		√			
Likely	c.G4297A	p.D1433N			√	√		√			
Uncertain	c.C4354T	p.R1452W				√		√			
Uncertain	-112_-107delCCCCGC					√		√			
Uncertain	-106C>G					√					√
Uncertain	-65G>C							√			
Uncertain	-23G>A					√					√
Uncertain	c.-82G>A					√					√
Likely	c.-6G>C				√	√					√
Uncertain	c.333-6C>A							√			

Uncertain	c.561+10 G>A			√	√			
Uncertain	c.1642+9 G>A			√	√			
Uncertain	c.1958+6 G>A			√	√			
Uncertain	c.1958+8 G>T			√	√			
Likely	c.2868-18_2868-7del		√	√	√			
Likely	c.2868-6C>G		√	√	√			
Uncertain	c.3083-6T>C				√			
Uncertain	c.3220+4 G>C			√	√			

Uncertain	c.3462+8 T>C			√	√			
Uncertain	c.4311+8 A>C			√	√			
Uncertain	c.*36G>C			√	√			
Uncertain	*7G>A			√				√
Likely	*14G>A		√	√				√
Uncertain	*16T>C			√				√
Uncertain	*46G>C			√	√			

† PM1 and PM5 evidence were from InterVar (http://wintervar.wglab.org/results.php).

‡ For sporadic cases, ACMG-AMP guidelines suggested using “Established risk allele” and “Likely risk allele” rather than “Pathogenic” and “Likely pathogenic” describing variants.

√ Variants meets the standard for corresponding criteria according to the ACMG-AMP guidelines.

Table S18. Evidentiary Summary for “Estabished risk” and “Likely risk” *WDR62* variants according to ACMG-AMP Guidelines

**c.2828dupA**	**p.Y943_S944delinsX**	**Estabished risk**
Very strong	PVS1	Nonsense variant
Moderate	PM2	Absent in 1000 G_2015aug, ESP6500SI-V2, and GnomAD
Supporting	PP3	The amino acid is highly conserved
**c.C3758A**	**p.S1253X**	**Estabished risk**
Very strong	PVS1	Nonsense variant.

Strong	PS4	Prevalence significantly increased in RVD over GnomAD allele counts for East Asian (OR: Inf, p adj.= 0.033).
Moderate	PM2	Absent in 1000 G_2015aug, ESP6500SI-V2, ExAC and GnomAD.
**c.G13C**	**p.G5R**	**Likely risk**
Strong	PS4	Prevalence significantly increased in DORV+PS over GnomAD allele counts for East Asian (OR: Inf, p adj.= 0.022).
Moderate	PM2	Absent in 1000 G_2015aug, ESP6500SI-V2, and extremely rare in GnomAD_exome (1.76E-05) and ExAC (5.83E-05)
**c.A875C**	**p.N292T**	**Likely risk**
Strong	PS3	Experimental functional studies demonstrate impaired heart development in 48 phf zebrafish, abnormal spindle assembly in cardiomyocytes.
Moderate	PM1	Located in a mutational hot spot and critical and WD40 repeat domain.
Supporting	PP3	Predicted to be "Damaging" by various algorithms and the nucleotide is highly conserved.
**c.G781A**	**p.G261S**	**Likely risk**
Strong	PS4	Prevalence significantly increased in DORV+PS over GnomAD allele counts for East Asian (OR: Inf, p adj.= 0.033).
Moderate	PM1	Extremely rare in GnomAD_EAS (5.44E-05).
Supporting	PP3	Predicted to be "Damaging" by various algorithms and the nucleotide is highly conserved.
**c.C1441T**	**p.R481W**	**Likely risk**
Strong	PS3	Experimental functional studies demonstrate impaired heart development in 48 phf zebrafish, abnormal spindle assembly in cardiomyocytes.
Moderate	PM1	Located in a mutational hot spot and critical and WD40 repeat domain.
	PM2	Absent in almost all population database except in GnomAD_exome where it is extremely rare (0.0004).
**c.C2515T**	**p.R839W**	**Likely risk**
Strong	PS4	Prevalence significantly increased in VSD over GnomAD allele counts for East Asian (OR: Inf, p adj.= 0.033).
Moderate	PM2	Absent in 1000 G_2015aug, ExAC and GnomAD_exome, and extremely rare in Exon Variant Server (7.70E-05) and GnomAD_genome (9.68E-05)
Supporting	PP3	Predicted to be "Damaging" by various algorithms and the nucleotide is highly conserved.
**c.C1669T**	**p.R557W**	**Likely risk**
Strong	PS3	Experimental functional studies demonstrate impaired heart development in 48 phf zebrafish, abnormal spindle assembly in cardiomyocytes.
Moderate	PM2	Absent in almost all population database except in GnomAD_exome where it is extremely rare (4.37E-06).
Supporting	PP3	The amino acid is highly conserved
**c.G1796C**	**p.C599S**	**Likely risk**

Strong	PS3	Experimental functional studies demonstrate impaired heart development in 48 phf zebrafish, abnormal spindle assembly in cardiomyocytes.
Moderate	PM1	Located in a mutational hot spot and critical and WD40 repeat domain.
**c.G3287A**	**p.R1096H**	**Likely risk**
Strong	PS3	Experimental functional studies demonstrate impaired heart development in 48 phf zebrafish, abnormal spindle assembly in cardiomyocytes.
Moderate	PM2	Extremely rare in ESP6500SI-V2 (0.0002).
**c.G4297A**	**p.D1433N**	**Likely risk**
Strong	PS4	Prevalence significantly increased in PA+VSD over GnomAD allele counts for East Asian (OR: Inf, p adj.= 0.033).
Moderate	PM2	Absent in almost all population database except in GnomAD_exome where it is extremely rare (1.22E-05).
**c.-6G>C**		**Likely risk**
Strong	PS4	Prevalence significantly increased in TOF and VSD over GnomAD allele counts for East Asian (OR: Inf, p adj.= 0.040).
Moderate	PM2	Absent in 1000 G_2015aug, ESP6500SI-V2, ExAC and GnomAD
**c.2868-18_2868-7del**		**Likely risk**
Strong	PS4	Prevalence significantly increased in VSD over GnomAD allele counts for East Asian (OR: Inf, p adj.= 0.0047).
Moderate	PM2	Absent in 1000 G_2015aug, ESP6500SI-V2, ExAC and GnomAD
Supporting	PP3	Splicing prediction algorithms predicted to break ESE and donor sites and create new splice sites
**c.2868-6C>G**		**Likely risk**
Strong	PS4	Prevalence significantly increased in VSD over GnomAD allele counts for East Asian (OR: Inf, p adj.= 0.0047).
Moderate	PM2	Absent in 1000 G_2015aug, ESP6500SI-V2, ExAC and GnomAD
Supporting	PP3	Splicing prediction algorithms predicted to break ESE and acceptor sites and create new splice site
***14G>A**		**Likely risk**
Strong	PS4	Prevalence significantly increased in PA+VSD over GnomAD allele counts for East Asian (OR: Inf, p adj.= 0.0047).
Moderate	PM2	Absent in 1000 G_2015aug, ESP6500SI-V2, ExAC and GnomAD

SUPPLEMENTAL FIGURES (S1-S8)

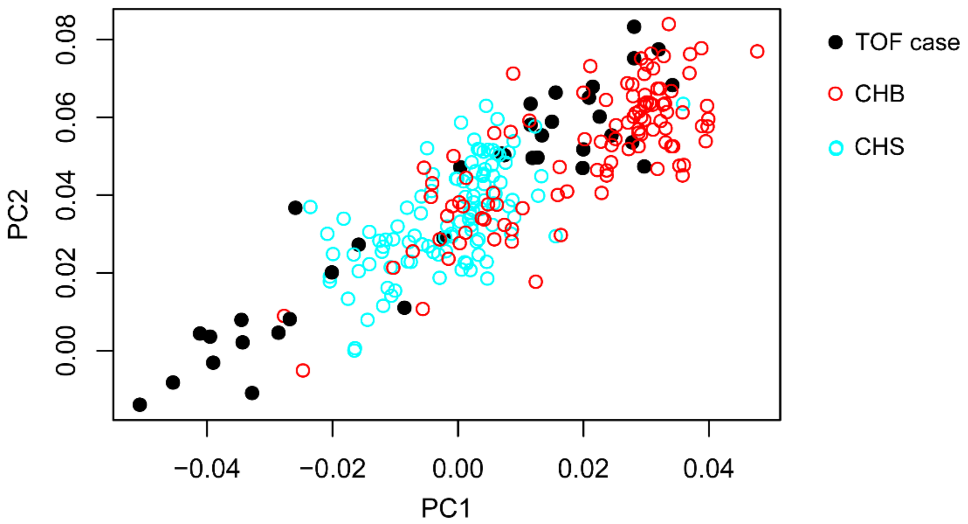

FIGURE S1 Principle components analysis (PCA) of ethnicity. The case (TOF cases indicated by black dots) and control from 1000 G (Han Chinese in Beijing [CHB] indicated by red circles and Southern Han Chinese [CHS] indicated by blue circles) were well matched.

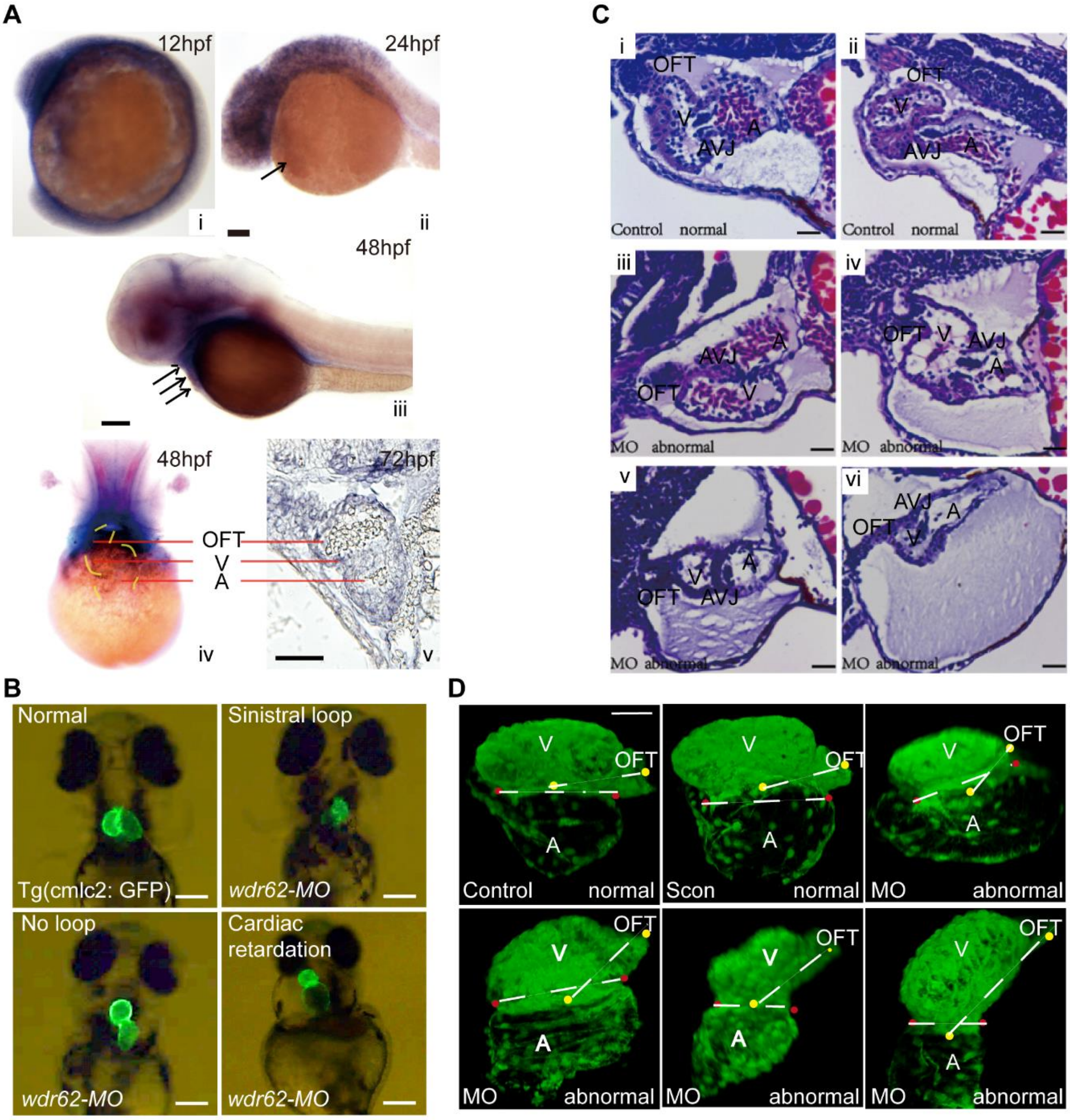

FIGURE S2 *wdr62* expression in zebrafish and the heart abnormalities of *wdr62*-MO.

(A) Whole-mount *in situ* hybridization by antisense *wdr62* probe in zebrafish at 12, 24, 48 and 72 h post-fertilization (hpf). At 12 hpf, *wdr62* was expressed in the whole body (i). At 24 hpf, the expression of *wdr62* was displayed in head and heart field (ii). At 48 hpf, *wdr62* was abundantly expressed in the heart and fins (iii). The magnified intact heart of 48 hpf zebrafish is shown in iv. Gene expression in the heart is indicated by the arrow. At 72 hpf, high expression

of *wdr62* in the heart, including ventricle, atrium and outflow tract, was observed through cardiac frozen section after hybridization and staining (v). Scale bar = 0.1 mm. (B**)** Representative cardiac morphology (cmlc2: GFP positive) of WT and *wdr62*-MO embryos at 72 hpf, which includes normal dextral loop, sinistral loop, no loop and cardiac growth delay. Scale bar = 0.2 mm. (C) H&E staining of zebrafish paraffin section at 72 hpf. i and ii show the normal heart of zebrafish in the control group; iii-vi show cardiac defects of *wdr62*-MO including thin wall of ventricle and atrium (iv-vi), smaller cardiac chamber (iv-vi), abnormal looping (iii-vi), lack of blood cell and pericardium edema (iv-vi). Scale bar = 50 µm. (D) Three dimensional reconstructions of heart (cmlc2: GFP positive) in WT or *wdr62*-MO zebrafish at 72 hpf. The degree of OFT rotation is shown by the angles between the atrium-ventricle interface (line between two red points) and OFT (line between two yellow points). The angles of *wdr62*-MO hearts were larger than the normal hearts in control groups. Scale bar = 50 µm. V denotes ventricle; A, atrium; OFT, outflow tract; AVJ, atrioventricular junction.

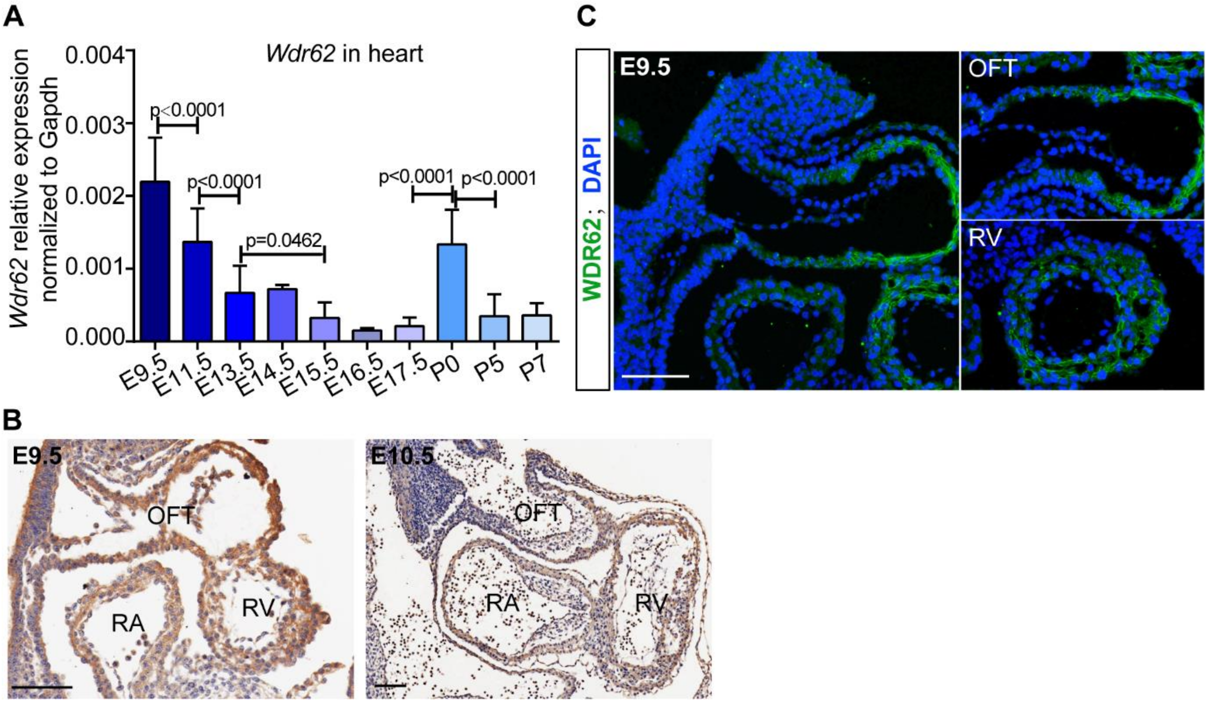

FIGURE S3 Spatio-temporal expression pattern of WDR62 in mouse hearts.

(A) Temporal expression pattern of *Wdr62* by qPCR in mouse heart from E9.5 to P7. (B) Distribution of WDR62 in cardiac tissues at E9.5 (image above) and E10.5 (image below) detected by immunohistochemistry. WDR62 was expressed widely in myocardial tissue of the heart; the brown color indicates WDR62 expression. WDR62 was expressed more in RV and OFT. Scale bar = 0.1 mm. (C) WDR62 expression (in green) by immunofluorescence in myocardium of E9.5 mice heart. The abundance of WDR62 was higher in RV and OFT. Scale bar = 0.1 mm.

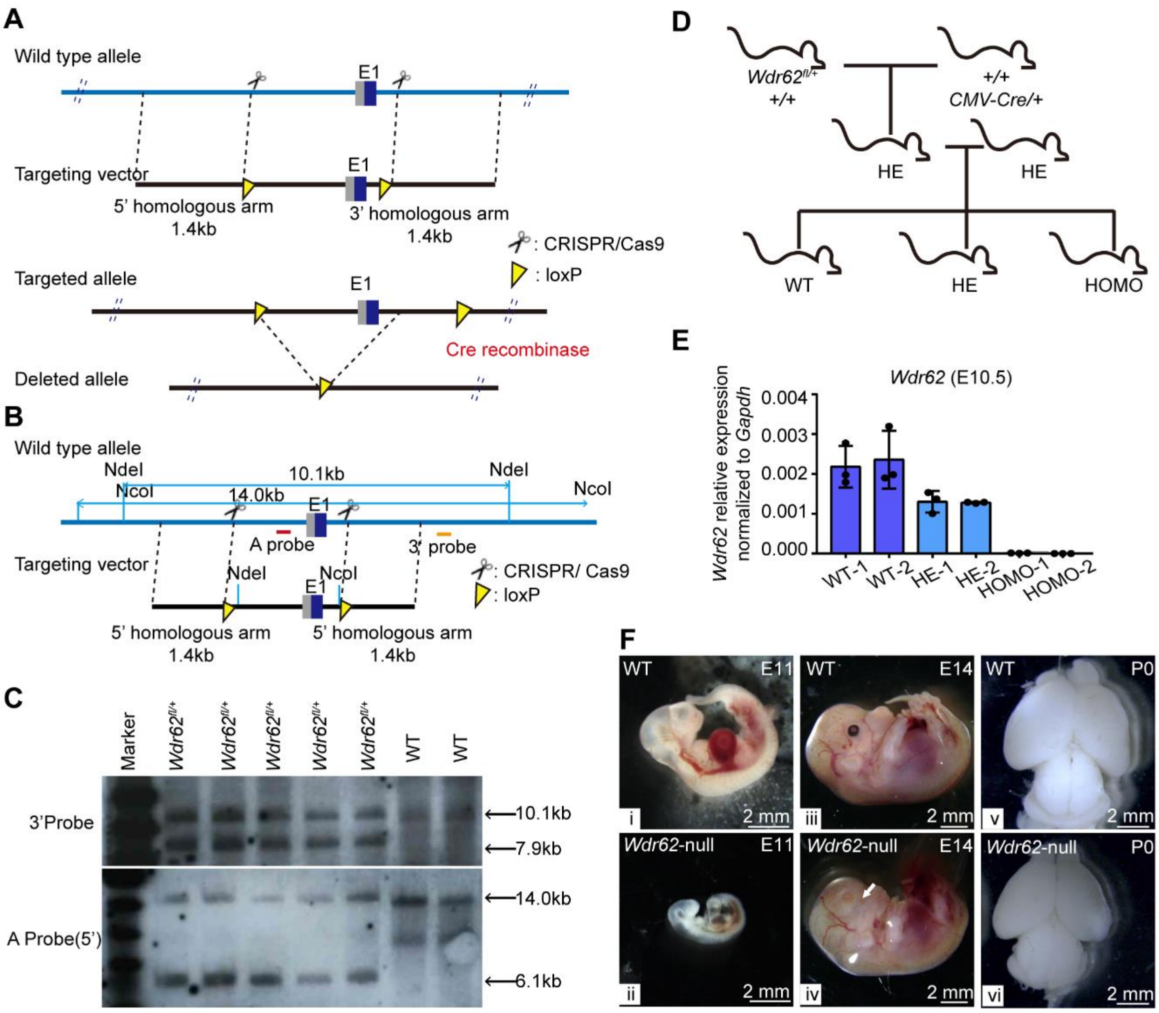

FIGURE S4 Generation and general defects of *Wdr62* knockout mice.

(A) Schematic of targeting strategy to generate floxed *Wdr62* allele as described in Supplemental Methods. (B, C**)** Southern blot analysis of *Wdr62*-flox mice. DNA was isolated from mouse tail and was digested with NdeI and hybridized with 3' Probe to detect whether correct recombination had occurred. Expected sizes for the correct recombined Wdr62-flox allele was 7.9 kb, wild-type allele was 10.1 kb. DNA was digested with NcoI and hybridized with A Probe to detect whether to detect whether there was random insertion. Expected sizes for the correct recombined Wdr62-flox allele was 14.0 kb, wild-type allele was 6.1 kb. Multiple bands will appear if there was random insertion. (D) Germline inactivation of *Wdr62* was achieved by crossing *Wdr62-flox* mice (*Wdr62*$^{fl/+}$) with *CMV-Cre*. The heterozygous *Wdr62*$^{fl/+}$; *CMV-Cre* mice (HE) were viable and were bred to obtain homozygous *Wdr62* knockout (HOMO) mice. (E) qPCR results of *Wdr62* mRNA extracted from embryonic tissue of WT and HOMO at E10.5. (F**)** Gross external morphology of WT and *Wdr62*-null mice. The developmental retardation (ii), coloboma or microphthalmia (iv) and microcephaly (vi) of *Wdr62*-null mice are shown at different developmental times. Scale bar = 2 mm.

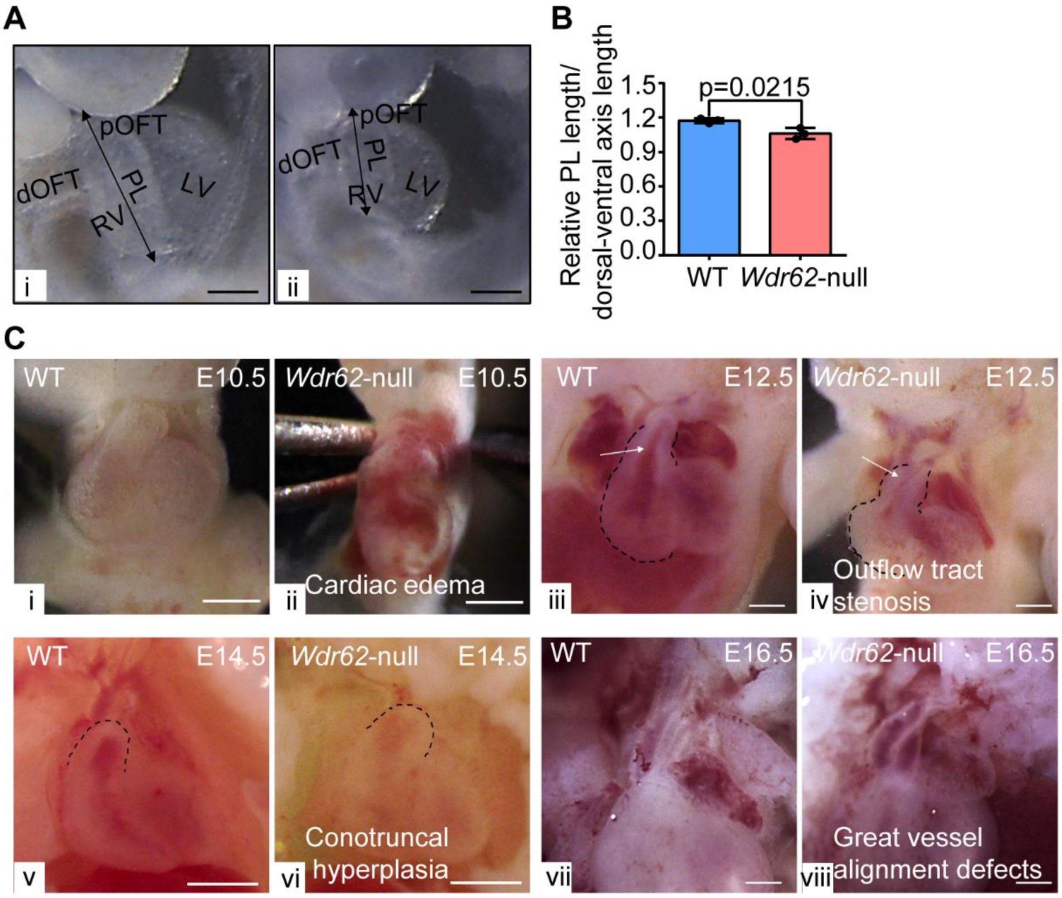

FIGURE S5 Heart phenotypes of *Wdr62*-null mice.

(A) Hearts of WT (i) and *Wdr62*-null (ii) mice at E9.5. The black lines drawn in the embryos show length. RV, right ventricle; LV, left ventricle; dOFT, distal OFT length; pOFT, proximal OFT; PL, proximal OFT+RV length. Scale bar = 0.2 mm. (B**)** Quantification analysis which revealed a significant decrease in PL length at E9.5 in *Wdr62*-null embryos compared with WT. The data are expressed as mean ± SD. (WT, n = 3; *Wdr62*-null, n = 3). (C) Representative cardiac morphology of WT and *Wdr62*-null embryos at different embryonic ages. Compared with WT, fetal hearts of *Wdr62*-null displayed cardiac edema at E10.5 (i and ii), outflow tract stenosis at E12.5 (iii and iv), swelling of the conotruncal region at E14.5 (v and vi), and great vessel alignment defects at E16.5 (vii and viii). Scale bar = 0.5 mm.

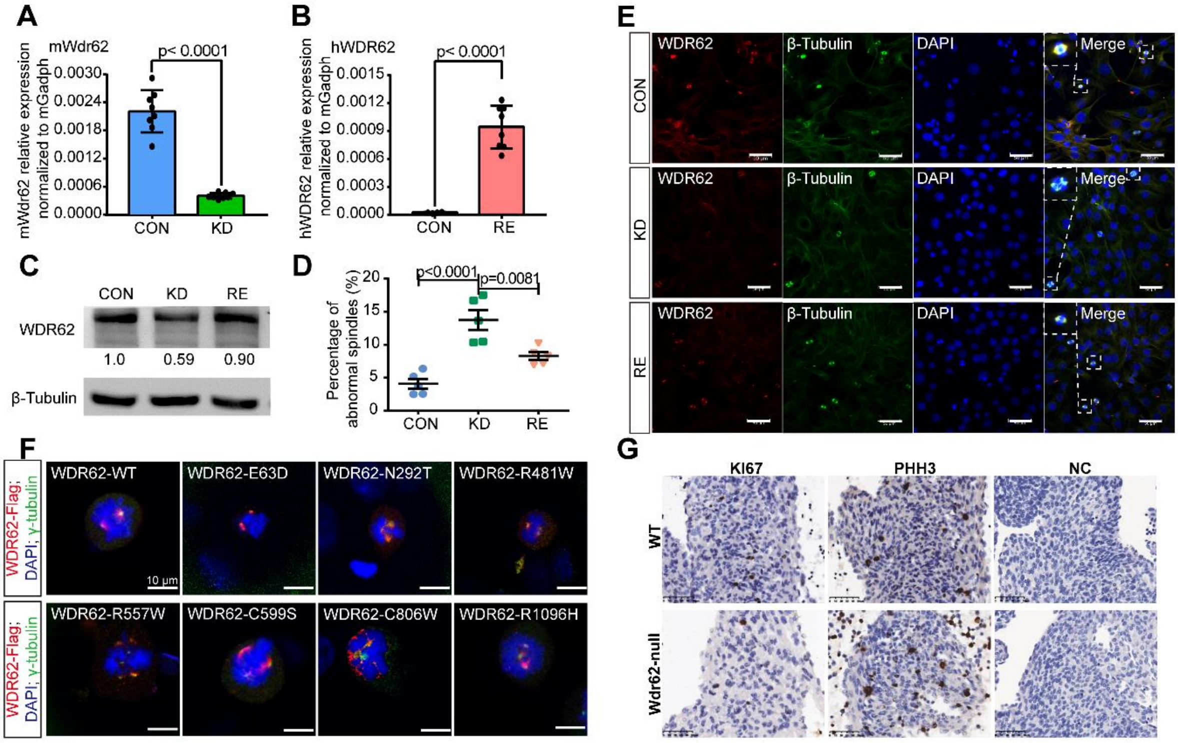

FIGURE S6 Construction of CON, KD and RE HL-1 cells and abnormal proliferation observed when WDR62 was deficient.

(A) Knockdown efficiency of KD group by qPCR. (B) Overexpression efficiency of RE group by qPCR. Overexpression was performed by stably transducing *hWDR62* in KD. (C) Western blot images showing the expression of WDR62 in three groups, which show the successful construction of WDR62 KD and RE. The captured amount of WDR62 was normalized to that of β-tubulin and the normalized values by greyscale scanning are indicated. (D) Percentage of cells with abnormal spindles in each group. Data are from five different views of each group and are expressed as mean ± SD. (E) Immunofluorescence images of HL-1 cells in CON, KD and RE groups. The mitotic cells are circled by dotted box and representative cells are magnified at the top left of merged images. HL-1 cells were stained to visualize spindle microtubule (β-tubulin; in green) and WDR62 (in red). Scale bar = 50 μm. (F). Seven WDR62

variants identified from CHD patients and WDR62-WT were overexpressed in HL-1 cells, respectively. Cells with multipolar spindles during mitotic prophase were found when WDR62 variants were overexpressed. HL-1 cells were stained to visualize chromosomes (DAPI; in blue), spindle poles (γ-tubulin; in green) and exogenous WDR62 (Flag; in red). Scale bar = 10 μm. (G) Ki67 (upper subpanel) and PHH3 (lower subpanel) positive cardiomyocytes in the interventricular septum (IVS) of mice at E14.5 are shown in brown. Slices from the same batch were only incubated with isotype matched secondary antibodies without binding with primary Ki67 or PHH3 antibodies. Nuclei of cardiomyocytes are shown in blue. Scale bar = 50 μm.

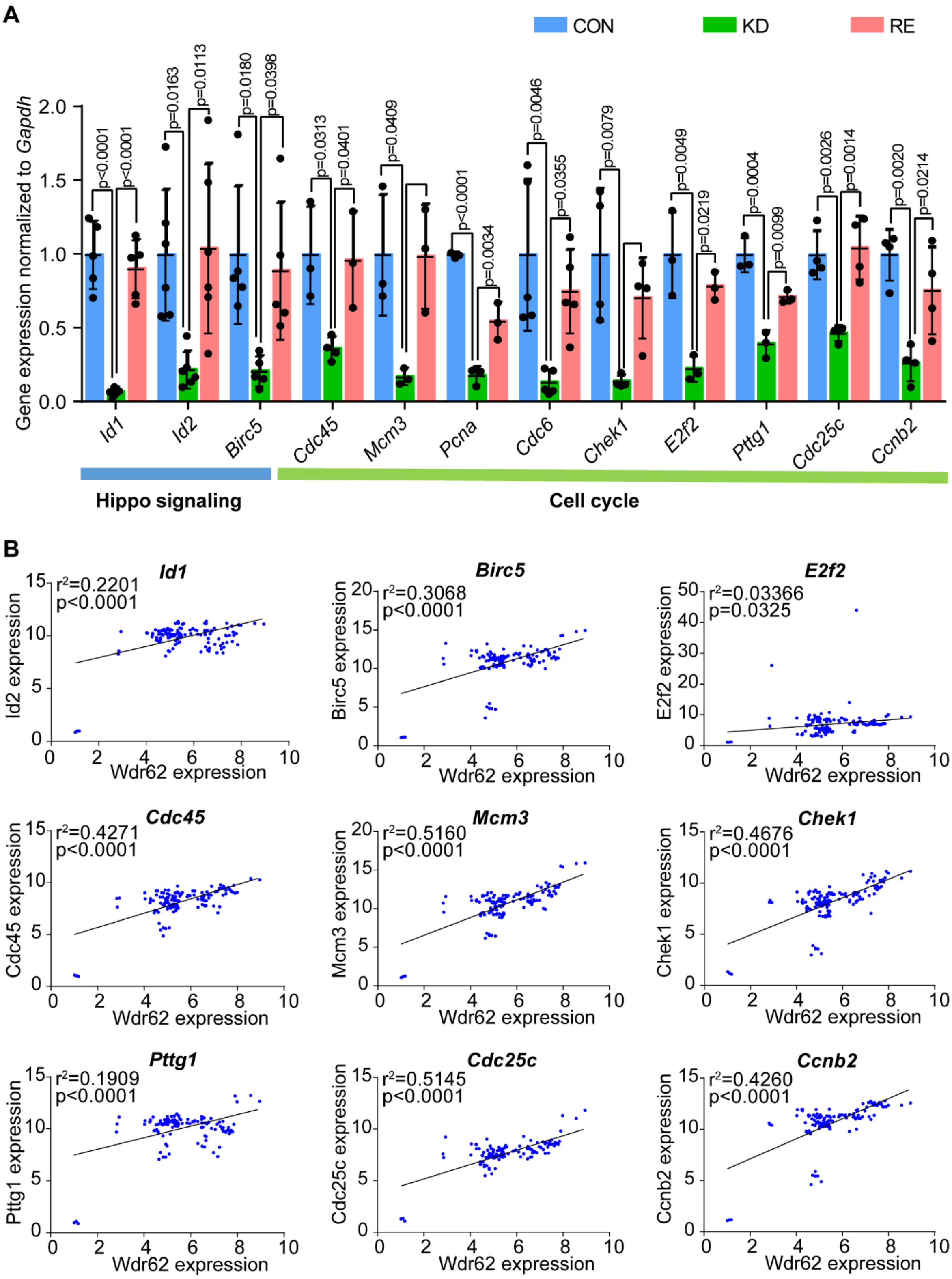

FIGURE S7 Downregulation of cell cycle and Hippo signaling caused by knockdown of WDR62.

(A) Expression of genes involved in cell cycle and Hippo signaling pathway in three groups of HL-1 cells including CON, KD and RE. The gene expression was decreased when WDR62 was knocked down, and partly recovered when hWDR62 was overexpressed. The data are expressed as mean ± SD of 3-7 experiments. (B) *Wdr62* is significantly correlated with selected genes in various heart tissues or cardiac stem cells from a wide range of developmental stages (n =136, Pearson correlation). The data series were obtained from microarray dataset of GEO database (GSE1479, GSE5298, GSE30593, GSE40260, GSE14906, GSE32078, GSE43197, GSE9124 and GSE70254).

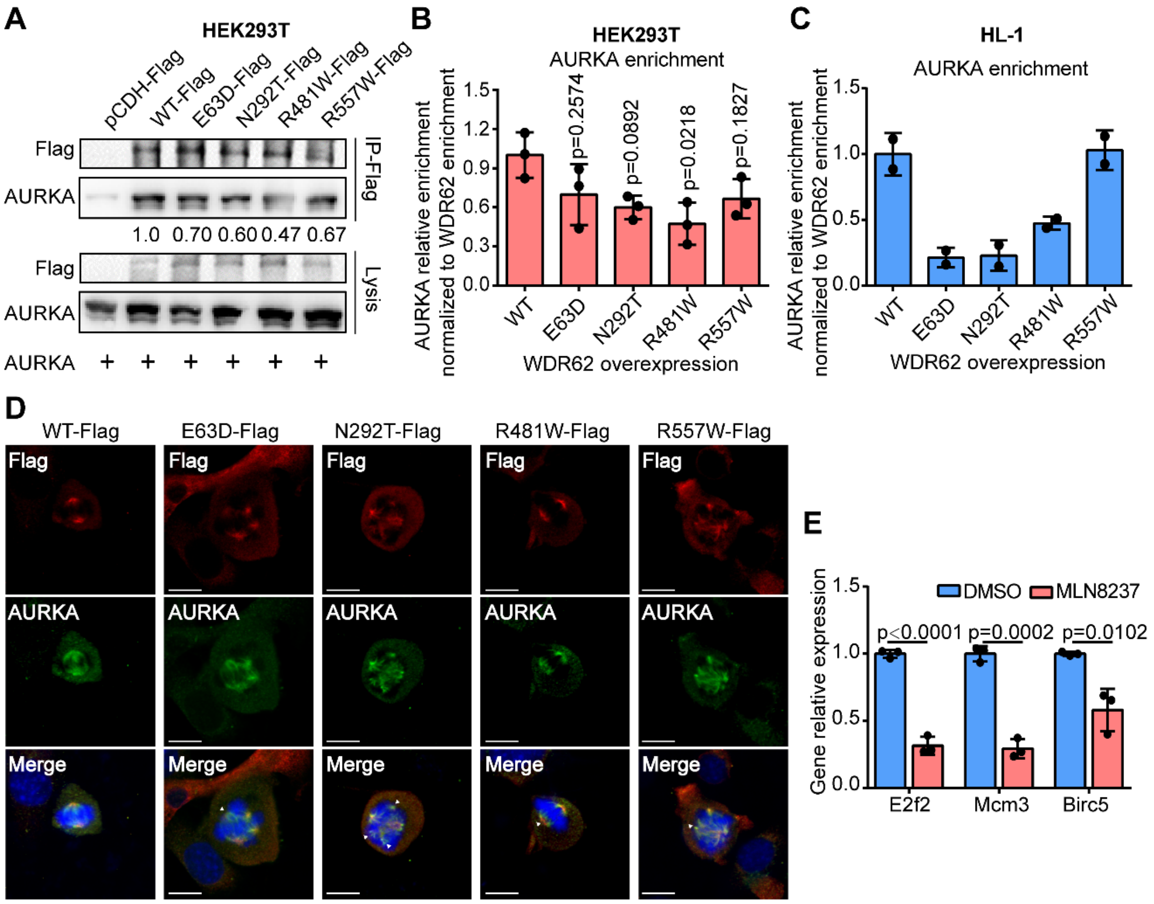

FIGURE S8 The harmful effects of WDR62 variants on WDR62-AURKA interaction.

(A) Co-IP of WDR62-WT and WDR62 variants with AURKA in HEK293T cells using Flag

antibodies, followed by probing with AURKA antibody to detect the AURKA interaction. The expression plasmid of AURKA was co-transfected with *WDR62*-WT or one of four *WDR62* variants in HEK293T cells. When *WDR62* variants were overexpressed, less AURKA was captured compared with overexpressing *WDR62*-WT. Representative images from three independent experiments are shown. The captured amount of AURKA in the representative images was normalized to that of Flag and the normalized values by greyscale scanning are indicated. (B, C) Semi-quantitative analysis: the captured amount of AURKA was normalized to that of Flag in HEK293T (B) and HL-1 (C) by greyscale scanning of strips. The data are expressed as mean ± SD of three (B) or four (C) independent experiments. *p*-values indicate comparison versus WT. (D**)** Co-localization of WDR62 and AURKA was observed when *WDR62*-WT was overexpressed in HL-1 cells (shown in yellow in merged panel). Overexpression of *WDR62* variants impaired the co-localization and microtubule assembly (indicated with white arrows). (E) HL-1 cells were treated with 500 nmol/l of MLN8237 or DMSO for 48 h. Expression of *E2f2*, *Mcm3* and *Birc5* were decreased when HL-1 cells were treated with MLN8237 as shown by qPCR.

REFERENCE

1. Ma Z, Xia W, Liu F*, et al.* SLC44A4 mutation causes autosomal dominant hereditary postlingual non-syndromic mid-frequency hearing loss. Human molecular genetics 2017;26:383-394.
2. Xiao D, Wang H, Hao L*, et al.* The roles of SMYD4 in epigenetic regulation of cardiac development in zebrafish. PLoS genetics 2018;14:e1007578.
3. Richards S, Aziz N, Bale S*, et al.* Standards and guidelines for the interpretation of sequence variants: a joint consensus recommendation of the American College of Medical Genetics and Genomics and the Association for Molecular Pathology. Genetics in medicine : official journal of the American College of Medical Genetics 2015;17:405-424.
4. Pollard KS, Hubisz MJ, Rosenbloom KR, Siepel A. Detection of nonneutral substitution

rates on mammalian phylogenies. Genome research 2010;20:110-121.
5. Davydov EV, Goode DL, Sirota M, Cooper GM, Sidow A, Batzoglou S. Identifying a high fraction of the human genome to be under selective constraint using GERP++. PLoS computational biology 2010;6:e1001025.
6. Siepel A, Bejerano G, Pedersen JS*, et al.* Evolutionarily conserved elements in vertebrate, insect, worm, and yeast genomes. Genome research 2005;15:1034-1050.
7. Shapiro MB, Senapathy P. RNA splice junctions of different classes of eukaryotes: sequence statistics and functional implications in gene expression. Nucleic acids research 1987;15:7155-7174.
8. Yeo G, Burge CB. Maximum entropy modeling of short sequence motifs with applications to RNA splicing signals. Journal of computational biology : a journal of computational molecular cell biology 2004;11:377-394.
9. Pertea M, Lin X, Salzberg SL. GeneSplicer: a new computational method for splice site prediction. Nucleic acids research 2001;29:1185-1190.
10. Reese MG, Eeckman FH, Kulp D, Haussler D. Improved splice site detection in Genie. Journal of computational biology : a journal of computational molecular cell biology 1997;4:311-323.
11. Cartegni L, Wang J, Zhu Z, Zhang MQ, Krainer AR. ESEfinder: A web resource to identify exonic splicing enhancers. Nucleic acids research 2003;31:3568-3571.
12. Fairbrother WG, Yeh RF, Sharp PA, Burge CB. Predictive identification of exonic splicing enhancers in human genes. Science (New York, NY) 2002;297:1007-1013.
13. Boyle AP, Hong EL, Hariharan M*, et al.* Annotation of functional variation in personal genomes using RegulomeDB. Genome research 2012;22:1790-1797.
14. Karimzadeh MR, Zarin M, Ehtesham N*, et al.* MicroRNA binding site polymorphism in inflammatory genes associated with colorectal cancer: literature review and bioinformatics analysis. Cancer gene therapy 2020;27:739-753.
15. Steri M, Idda ML, Whalen MB, Orrù V. Genetic variants in mRNA untranslated regions. Wiley interdisciplinary reviews RNA 2018;9:e1474.
